# Supplementary material for: An agent-based model to simulate the transmission dynamics of bloodborne pathogens within hospitals
Source: PLoS Comput Biol. 2025 Feb 24;21(2):e1012850. doi: 10.1371/journal.pcbi.1012850 (PMC11882061; doi:10.1371/journal.pcbi.1012850)
Supplement: S1 Table — (DOCX) [file pcbi.1012850.s001.docx]

**Table S1.** Ward ID and names

| **Ward ID** | **Ward name** | **Initial prevalence (HCV)** | **Initial prevalence (HBV)** |
| --- | --- | --- | --- |
| 1 | GIT & Endoscopy | 0% | 0% |
| 2 | Endocrinology | 50% | 11.5% |
| 3 | Immunology & Allergy | 0% | 0% |
| 4 | Hematology | 0% | 7.1% |
| 5 | Rheumatology | 0% | 9.3% |
| 6 | Geriatric | 0% | 0% |
| 7 | Chest | 0% | 6% |
| 8 | Nephrology | 0% | 3% |
| 9 | Cardiology | 0% | 13% |
| 11 | Neurology | 0% | 14% |
| 12 | Tropical medicine | 51.8% | 8.4% |
| 13 | Dermatology | 0% | 0% |
| 14 | Ophtalmology | 13.6% | 7.5% |
| 16 | ICU | 0% | 0% |
| 17 | Orthopedics | 3.1% | 7.4% |
| 18 | General surgery | 4.2% | 7.4% |
| 20 | Neurosurgery | 25% | 9% |
| 21 | Urosurgery | 100% | 6.3% |
| 22 | Burn | 0% | 0% |
| 23 | ENT | 0% | 0% |
| 24 | Plastic surgery | 5.3% | 6.2% |
| 25 | Operating room (OR) | 0% | 0% |
| 26 | Radiology | 0% | 0% |
| 27 | Clinical pathology | 0% | 3% |
| 28 | Endoscopy | 0% | 0% |
| 29 | Physical medicine | 0% | 9.5% |
| 30 | Emergency room (ER) | 9% | 7.3% |
| 31 | Emergency room ICU | 0% | 0% |
