# Supplementary material for: An agent-based model to simulate the transmission dynamics of bloodborne pathogens within hospitals
Source: PLoS Comput Biol. 2025 Feb 24;21(2):e1012850. doi: 10.1371/journal.pcbi.1012850 (PMC11882061; doi:10.1371/journal.pcbi.1012850)
Supplement: S3 Table — Each cell provides the proportion of patients moving from ward i (in line) to ward j (in column) at each time-step. (DOCX) [file pcbi.1012850.s003.docx]

**Table S3.** Transition matrix for patients hospitalized in the surgery department. Each cell provides the proportion of patients moving from ward i (in line) to ward j (in column) at each time-step.

|  | **1** | **2** | **3** | **4** | **5** | **6** | **7** | **8** | **9** | **11** | **12** | **13** | **14** | **16** | **17** |  | **18** | **20** | **21** | **22** | **23** | **24** | **25** | **26** | **27** | **28** | **29** | **30** | **31** | **out** |
| --- | --- | --- | --- | --- | --- | --- | --- | --- | --- | --- | --- | --- | --- | --- | --- | --- | --- | --- | --- | --- | --- | --- | --- | --- | --- | --- | --- | --- | --- | --- |
| **1** | 0 | 0 | 0 | 0 | 0 | 0 | 0 | 0 | 0 | 0 | 0 | 0 | 0 | 0 | 0 |  | 0 | 0 | 0 | 0 | 0 | 0 | 0 | 0 | 0 | 0 | 0 | 0 | 0 | 0 |
| **2** | 0 | 0 | 0 | 0 | 0 | 0 | 0 | 0 | 0 | 0 | 0 | 0 | 0 | 0 | 0 |  | 0 | 0 | 0 | 0 | 0 | 0 | 0 | 0 | 0 | 0 | 0 | 0 | 0 | 0 |
| **3** | 0 | 0 | 0 | 0 | 0 | 0 | 0 | 0 | 0 | 0 | 0 | 0 | 0 | 0 | 0 |  | 0 | 0 | 0 | 0 | 0 | 0 | 0 | 0 | 0 | 0 | 0 | 0 | 0 | 0 |
| **4** | 0 | 0 | 0 | 0 | 0 | 0 | 0 | 0 | 0 | 0 | 0 | 0 | 0 | 0 | 0 |  | 0 | 0 | 0 | 0 | 0 | 0 | 0 | 0 | 0 | 0 | 0 | 0 | 0 | 0 |
| **5** | 0 | 0 | 0 | 0 | 0 | 0 | 0 | 0 | 0 | 0 | 0 | 0 | 0 | 0 | 0 |  | 0 | 0 | 0 | 0 | 0 | 0 | 0 | 0 | 0 | 0 | 0 | 0 | 0 | 0 |
| **6** | 0 | 0 | 0 | 0 | 0 | 0 | 0 | 0 | 0 | 0 | 0 | 0 | 0 | 0 | 0 |  | 0 | 0 | 0 | 0 | 0 | 0 | 0 | 0 | 0 | 0 | 0 | 0 | 0 | 0 |
| **7** | 0 | 0 | 0 | 0 | 0 | 0 | 0 | 0 | 0 | 0 | 0 | 0 | 0 | 0 | 0 |  | 0 | 0 | 0 | 0 | 0 | 0 | 0 | 0 | 0 | 0 | 0 | 0 | 0 | 0 |
| **8** | 0 | 0 | 0 | 0 | 0 | 0 | 0 | 0 | 0 | 0 | 0 | 0 | 0 | 0 | 0 |  | 0 | 0 | 0 | 0 | 0 | 0 | 0 | 0 | 0 | 0 | 0 | 0 | 0 | 0 |
| **9** | 0 | 0 | 0 | 0 | 0 | 0 | 0 | 0 | 0,875 | 0 | 0 | 0 | 0 | 0 | 0,09375 |  | 0,03125 | 0 | 0 | 0 | 0 | 0 | 0 | 0 | 0 | 0 | 0 | 0 | 0 | 0 |
| **11** | 0 | 0 | 0 | 0 | 0 | 0 | 0 | 0 | 0 | 0 | 0 | 0 | 0 | 0 | 0 |  | 0 | 0 | 0 | 0 | 0 | 0 | 0 | 0 | 0 | 0 | 0 | 0 | 0 | 0 |
| **12** | 0 | 0 | 0 | 0 | 0 | 0 | 0 | 0 | 0 | 0 | 0 | 0 | 0 | 0 | 0 |  | 0 | 0 | 0 | 0 | 0 | 0 | 0 | 0 | 0 | 0 | 0 | 0 | 0 | 0 |
| **13** | 0 | 0 | 0 | 0 | 0 | 0 | 0 | 0 | 0 | 0 | 0 | 0 | 0 | 0 | 0 |  | 0 | 0 | 0 | 0 | 0 | 0 | 0 | 0 | 0 | 0 | 0 | 0 | 0 | 0 |
| **14** | 0 | 0 | 0 | 0 | 0 | 0 | 0 | 0 | 0 | 0 | 0 | 0 | 0 | 0 | 0 |  | 0 | 0 | 0 | 0 | 0 | 0 | 0 | 0 | 0 | 0 | 0 | 0 | 0 | 0 |
| **16** | 0 | 0 | 0 | 0 | 0 | 0 | 0 | 0 | 0 | 0 | 0 | 0 | 0 | 0,998093 | 0,000636 |  | 0,000318 | 0,000636 | 0 | 0 | 0 | 0 | 0 | 0,000318 | 0 | 0 | 0 | 0 | 0 | 0 |
| **17** | 0 | 0 | 0 | 0 | 0 | 0 | 0 | 0 | 3,50E-05 | 0 | 0 | 0 | 0 | 0 | 0,997379 |  | 0,000104 | 2,30E-05 | 0 | 0 | 0 | 2,30E-05 | 0,000543 | 0,001028 | 0,000115 | 0 | 0 | 5,80E-05 | 0 | 0,000693 |
| **18** | 0 | 0 | 0 | 0 | 0 | 0 | 0 | 0 | 9,00E-06 | 0 | 0 | 0 | 0 | 0 | 4,30E-05 |  | 0,996972 | 1,70E-05 | 9,00E-06 | 0 | 0 | 1,70E-05 | 0,000981 | 0,00037 | 0,000146 | 1,70E-05 | 0 | 0 | 0 | 0,001419 |
| **20** | 0 | 0 | 0 | 0 | 0 | 0 | 0 | 0 | 0 | 0 | 0 | 0 | 0 | 0,000107 | 0,000107 |  | 0 | 0,997964 | 0 | 0 | 0,000107 | 0 | 0,000429 | 0,000536 | 0,000107 | 0 | 0 | 0 | 0 | 0,000643 |
| **21** | 0 | 0 | 0 | 0 | 0 | 0 | 0 | 0 | 0 | 0 | 0 | 0 | 0 | 0 | 0 |  | 0 | 0 | 0,997276 | 0 | 0 | 0 | 0,000227 | 0,000908 | 0 | 0 | 0 | 0 | 0 | 0,001589 |
| **22** | 0 | 0 | 0 | 0 | 0 | 0 | 0 | 0 | 0 | 0 | 0 | 0 | 0 | 0 | 0,035714 |  | 0 | 0 | 0 | 0,928571 | 0 | 0 | 0 | 0,035714 | 0 | 0 | 0 | 0 | 0 | 0 |
| **23** | 0 | 0 | 0 | 0 | 0 | 0 | 0 | 0 | 0 | 0 | 0 | 0 | 0 | 0 | 0 |  | 0,047619 | 0 | 0 | 0 | 0,952381 | 0 | 0 | 0 | 0 | 0 | 0 | 0 | 0 | 0 |
| **24** | 0 | 0 | 0 | 0 | 0 | 0 | 0 | 0 | 0 | 0 | 0 | 0 | 0 | 0 | 4,60E-05 |  | 4,60E-05 | 0 | 0 | 0 | 0 | 0,996007 | 0,001393 | 0,000325 | 0,000557 | 0 | 0 | 0 | 0 | 0,001625 |
| **25** | 0 | 0 | 0 | 0 | 0 | 0 | 0 | 0 | 0 | 0 | 0 | 0 | 0 | 0,000421 | 0,006453 |  | 0,016552 | 0,000421 | 0,00014 | 0 | 0 | 0,004208 | 0,971665 | 0 | 0 | 0 | 0 | 0 | 0 | 0,00014 |
| **26** | 0 | 0 | 0 | 0 | 0 | 0 | 0 | 0 | 0 | 0 | 0 | 0 | 0 | 0,000759 | 0,040971 |  | 0,021624 | 0,001897 | 0,001517 | 0,000379 | 0 | 0,00569 | 0,001138 | 0,901745 | 0,000759 | 0 | 0 | 0,02352 | 0 | 0 |
| **27** | 0 | 0 | 0 | 0 | 0 | 0 | 0 | 0 | 0 | 0 | 0 | 0 | 0 | 0 | 0,018519 |  | 0,034722 | 0 | 0 | 0,002315 | 0 | 0,020833 | 0 | 0,030093 | 0,837963 | 0 | 0 | 0,055556 | 0 | 0 |
| **28** | 0 | 0 | 0 | 0 | 0 | 0 | 0 | 0 | 0 | 0 | 0 | 0 | 0 | 0 | 0 |  | 0,022989 | 0 | 0 | 0 | 0 | 0 | 0 | 0 | 0 | 0,977011 | 0 | 0 | 0 | 0 |
| **29** | 0 | 0 | 0 | 0 | 0 | 0 | 0 | 0 | 0 | 0 | 0 | 0 | 0 | 0 | 0 |  | 0 | 0 | 0 | 0 | 0 | 0 | 0 | 0 | 0 | 0 | 0 | 0 | 0 | 0 |
| **30** | 0 | 0 | 0 | 0 | 0 | 0 | 0 | 0 | 0 | 0 | 0 | 0 | 0 | 0 | 0,004843 |  | 0,017528 | 0,000231 | 0,000461 | 0 | 0 | 0,002076 | 0,000692 | 0,02214 | 0,006227 | 0 | 0 | 0,945803 | 0 | 0 |
| **31** | 0 | 0 | 0 | 0 | 0 | 0 | 0 | 0 | 0 | 0 | 0 | 0 | 0 | 0 | 0 |  | 0 | 0 | 0 | 0 | 0 | 0 | 0 | 0 | 0 | 0 | 0 | 0 | 0 | 0 |
| **out** | 0 | 0 | 0 | 0 | 0 | 0 | 0 | 0 | 0 | 0 | 0 | 0 | 0 | 0 | 0 |  | 0 | 0 | 0 | 0 | 0 | 0 | 0 | 0 | 0 | 0 | 0 | 0 | 0 | 1 |
