# Supplementary material for: An agent-based model to simulate the transmission dynamics of bloodborne pathogens within hospitals
Source: PLoS Comput Biol. 2025 Feb 24;21(2):e1012850. doi: 10.1371/journal.pcbi.1012850 (PMC11882061; doi:10.1371/journal.pcbi.1012850)
Supplement: S4 Table — Each cell provides the proportion of patients moving from ward i (in line) to ward j (in column) at each time-step. (DOCX) [file pcbi.1012850.s004.docx]

| **1** | 0,997737 | 0,000112 | 2,80E-05 | 0 | 0 | 0,000112 | 0 | 0,000251 | 0,000168 | 0 | 0 | 0 | 5,60E-05 | 0 | 0 | 0 | 0 | 0 | 0 | 0 | 0 | 0 | 0,000698 | 0 | 0,00014 | 0 | 0 | 2,80E-05 | 0,000671 |
| --- | --- | --- | --- | --- | --- | --- | --- | --- | --- | --- | --- | --- | --- | --- | --- | --- | --- | --- | --- | --- | --- | --- | --- | --- | --- | --- | --- | --- | --- |
| **2** | 6,20E-05 | 0,997485 | 6,20E-05 | 0 | 4,10E-05 | 0 | 2,10E-05 | 0,000144 | 0,000247 | 8,20E-05 | 4,10E-05 | 0 | 2,10E-05 | 0 | 0 | 2,10E-05 | 0 | 0 | 0 | 0 | 0 | 0 | 0,000928 | 0 | 0,000124 | 0 | 2,10E-05 | 0 | 0,000701 |
| **3** | 2,20E-05 | 8,80E-05 | 0,997426 | 2,20E-05 | 4,40E-05 | 4,40E-05 | 0 | 0,000242 | 0,000396 | 0 | 0 | 4,40E-05 | 6,60E-05 | 0 | 0 | 4,40E-05 | 0 | 0 | 0 | 0 | 0 | 0 | 0,000902 | 0 | 6,60E-05 | 0 | 6,60E-05 | 2,20E-05 | 0,000506 |
| **4** | 0 | 0 | 5,40E-05 | 0,998757 | 0 | 0 | 0 | 0 | 0 | 0 | 0 | 0 | 0 | 0 | 0 | 0 | 0 | 0 | 0 | 0 | 0 | 0 | 0,000756 | 0 | 0 | 0 | 0 | 5,40E-05 | 0,000378 |
| **5** | 0 | 5,10E-05 | 5,10E-05 | 0 | 0,997434 | 0 | 0 | 0,000154 | 0,000462 | 5,10E-05 | 0 | 0 | 0,000205 | 0 | 0 | 0 | 0 | 0 | 0 | 0 | 0 | 5,10E-05 | 0,000873 | 0 | 0 | 0 | 0 | 0 | 0,000667 |
| **6** | 0,000338 | 8,40E-05 | 0 | 0 | 0 | 0,998649 | 0 | 0,000169 | 0,000169 | 0 | 0 | 0 | 0 | 0 | 0 | 0 | 0 | 0 | 0 | 0 | 0 | 0 | 0,000253 | 0 | 8,40E-05 | 0 | 0 | 0 | 0,000253 |
| **7** | 0 | 0 | 0 | 0 | 0 | 0 | 0,998868 | 0 | 0,000113 | 0 | 0 | 0 | 0,000113 | 0 | 0 | 0 | 0 | 0 | 0 | 0 | 0 | 0 | 0,000566 | 0 | 0 | 0 | 0 | 0 | 0,00034 |
| **8** | 0,000184 | 0,00021 | 0,000315 | 2,60E-05 | 7,90E-05 | 5,20E-05 | 0 | 0,99701 | 0,000184 | 0 | 0,000184 | 0 | 5,20E-05 | 0 | 0 | 5,20E-05 | 0 | 0 | 0 | 7,90E-05 | 0 | 0 | 0,000865 | 0 | 5,20E-05 | 0 | 5,20E-05 | 5,20E-05 | 0,000551 |
| **9** | 0,000874 | 0,000874 | 0,001661 | 0,000175 | 0,000874 | 0,000175 | 0,000437 | 0,000787 | 0,988372 | 0 | 0,000612 | 0 | 0,003147 | 0 | 0 | 0 | 0 | 0 | 0 | 0 | 0 | 0 | 0,001049 | 0 | 0,000175 | 0 | 0 | 0 | 0,000787 |
| **11** | 0 | 0,000427 | 0 | 0 | 0,000142 | 0 | 0 | 0 | 0 | 0,998151 | 0 | 0 | 0 | 0 | 0 | 0 | 0 | 0 | 0 | 0 | 0 | 0 | 0,000569 | 0 | 0 | 0 | 0 | 0 | 0,000711 |
| **12** | 2,50E-05 | 2,50E-05 | 0 | 0 | 0 | 0 | 0 | 0,000175 | 0,00015 | 0 | 0,996926 | 2,50E-05 | 0 | 0 | 0 | 0 | 0 | 0 | 0 | 0 | 0 | 0 | 0,001849 | 0 | 7,50E-05 | 0 | 0 | 2,50E-05 | 0,000725 |
| **13** | 0 | 0 | 0,0625 | 0 | 0 | 0 | 0 | 0 | 0,0625 | 0 | 0,0625 | 0,8125 | 0 | 0 | 0 | 0 | 0 | 0 | 0 | 0 | 0 | 0 | 0 | 0 | 0 | 0 | 0 | 0 | 0 |
| **14** | 2,30E-05 | 2,30E-05 | 3,40E-05 | 0 | 5,70E-05 | 0 | 1,10E-05 | 4,60E-05 | 0,000402 | 0 | 1,10E-05 | 0 | 0,998794 | 0 | 0 | 0 | 0 | 0 | 0 | 0 | 0 | 0 | 5,70E-05 | 0 | 1,10E-05 | 0 | 1,10E-05 | 0 | 0,000517 |
| **16** | 0 | 0 | 0 | 0 | 0 | 0 | 0 | 0 | 0 | 0 | 0 | 0 | 0 | 0 | 0 | 0 | 0 | 0 | 0 | 0 | 0 | 0 | 0 | 0 | 0 | 0 | 0 | 0 | 0 |
| **17** | 0 | 0 | 0 | 0 | 0 | 0 | 0 | 0 | 0 | 0 | 0 | 0 | 0 | 0 | 0 | 0 | 0 | 0 | 0 | 0 | 0 | 0 | 0 | 0 | 0 | 0 | 0 | 0 | 0 |
| **18** | 0 | 0,020833 | 0,010417 | 0 | 0 | 0 | 0 | 0,010417 | 0 | 0 | 0 | 0 | 0,010417 | 0 | 0 | 0,927083 | 0 | 0 | 0 | 0 | 0 | 0,010417 | 0 | 0 | 0 | 0 | 0,010417 | 0 | 0 |
| **20** | 0 | 0 | 0 | 0 | 0 | 0 | 0 | 0 | 0 | 0 | 0 | 0 | 0 | 0 | 0 | 0 | 0 | 0 | 0 | 0 | 0 | 0 | 0 | 0 | 0 | 0 | 0 | 0 | 0 |
| **21** | 0 | 0 | 0 | 0 | 0 | 0 | 0 | 0 | 0 | 0 | 0 | 0 | 0 | 0 | 0 | 0 | 0 | 0 | 0 | 0 | 0 | 0 | 0 | 0 | 0 | 0 | 0 | 0 | 0 |
| **22** | 0 | 0 | 0 | 0 | 0 | 0 | 0 | 0 | 0 | 0 | 0 | 0 | 0 | 0 | 0 | 0 | 0 | 0 | 0 | 0 | 0 | 0 | 0 | 0 | 0 | 0 | 0 | 0 | 0 |
| **23** | 0 | 0 | 0 | 0 | 0 | 0 | 0 | 0,040541 | 0 | 0 | 0 | 0 | 0 | 0 | 0 | 0 | 0 | 0 | 0 | 0,959459 | 0 | 0 | 0 | 0 | 0 | 0 | 0 | 0 | 0 |
| **24** | 0 | 0 | 0 | 0 | 0 | 0 | 0 | 0 | 0 | 0 | 0 | 0 | 0 | 0 | 0 | 0 | 0 | 0 | 0 | 0 | 0 | 0 | 0 | 0 | 0 | 0 | 0 | 0 | 0 |
| **25** | 0 | 0 | 0 | 0 | 0,013889 | 0 | 0 | 0 | 0 | 0 | 0 | 0 | 0 | 0 | 0 | 0,013889 | 0 | 0 | 0 | 0 | 0 | 0,972222 | 0 | 0 | 0 | 0 | 0 | 0 | 0 |
| **26** | 0,005892 | 0,012319 | 0,011784 | 0,003214 | 0,004017 | 0,000803 | 0,00241 | 0,008034 | 0,00616 | 0,001339 | 0,019014 | 0 | 0,001875 | 0 | 0 | 0 | 0 | 0 | 0 | 0 | 0 | 0 | 0,905731 | 0 | 0,000268 | 0,001339 | 0,015801 | 0 | 0 |
| **27** | 0 | 0 | 0 | 0 | 0 | 0 | 0 | 0 | 0 | 0 | 0 | 0 | 0 | 0 | 0 | 0 | 0 | 0 | 0 | 0 | 0 | 0 | 0 | 0 | 0 | 0 | 0 | 0 | 0 |
| **28** | 0,014577 | 0,020408 | 0,011662 | 0 | 0 | 0,002915 | 0 | 0,008746 | 0 | 0 | 0,011662 | 0 | 0,002915 | 0 | 0 | 0 | 0 | 0 | 0 | 0 | 0 | 0 | 0 | 0 | 0,915452 | 0 | 0,005831 | 0,005831 | 0 |
| **29** | 0 | 0 | 0 | 0 | 0 | 0 | 0 | 0 | 0 | 0 | 0 | 0 | 0 | 0 | 0 | 0 | 0 | 0 | 0 | 0 | 0 | 0 | 0,001572 | 0 | 0 | 0,997799 | 0 | 0 | 0,000629 |
| **30** | 0,002532 | 0,003271 | 0,002849 | 0 | 0,000739 | 0,000211 | 0,000317 | 0,002427 | 0,001161 | 0,000106 | 0,000317 | 0 | 0,000211 | 0 | 0 | 0 | 0 | 0 | 0 | 0 | 0 | 0 | 0,006753 | 0 | 0,000422 | 0 | 0,978685 | 0 | 0 |
| **31** | 0,000201 | 0 | 0 | 0 | 0 | 0 | 0 | 1,00E-04 | 0 | 0 | 0 | 0 | 0 | 0 | 0 | 0 | 0 | 0 | 0 | 0 | 0 | 0 | 0 | 0 | 1,00E-04 | 0 | 0 | 0,999197 | 0,000402 |
| **out** | 0 | 0 | 0 | 0 | 0 | 0 | 0 | 0 | 0 | 0 | 0 | 0 | 0 | 0 | 0 | 0 | 0 | 0 | 0 | 0 | 0 | 0 | 0 | 0 | 0 | 0 | 0 | 0 | 1 |

**Table S4.** Transition matrix for patients hospitalized in the internal medicine department. Each cell provides the proportion of patients moving from ward i (in line) to ward j (in column) at each time-step.
