# Supplementary material for: An agent-based model to simulate the transmission dynamics of bloodborne pathogens within hospitals
Source: PLoS Comput Biol. 2025 Feb 24;21(2):e1012850. doi: 10.1371/journal.pcbi.1012850 (PMC11882061; doi:10.1371/journal.pcbi.1012850)
Supplement: S5 Table — Each cell provides the probability of undergoing procedure j (in column) in ward I (in line) at each time-step. (DOCX) [file pcbi.1012850.s005.docx]

**Table S5.** Ward-specific probabilities of undergoing each of the procedures in the surgery department. Each cell provides the probability of undergoing procedure j (in column) in ward I (in line) at each time-step.

|  | **surgery** | **intravenous** | **sutures** | **blood_transfusion** | **blood_sample** | **injection** | **endoscopy** | **gastric_lavage** | **cardiac_catheter** | **dialysis** | **wound_dressing** | **bloodglucose** | **endotrachealintu** | **drainagecatheter** | **other_invproc** | **no_proc** |
| --- | --- | --- | --- | --- | --- | --- | --- | --- | --- | --- | --- | --- | --- | --- | --- | --- |
| **1** | 0 | 0 | 0 | 0 | 0 | 0 | 0 | 0 | 0 | 0 | 0 | 0 | 0 | 0 | 0 | 1 |
| **2** | 0 | 0 | 0 | 0 | 0 | 0 | 0 | 0 | 0 | 0 | 0 | 0 | 0 | 0 | 0 | 1 |
| **3** | 0 | 0 | 0 | 0 | 0 | 0 | 0 | 0 | 0 | 0 | 0 | 0 | 0 | 0 | 0 | 1 |
| **4** | 0 | 0 | 0 | 0 | 0 | 0 | 0 | 0 | 0 | 0 | 0 | 0 | 0 | 0 | 0 | 1 |
| **5** | 0 | 0 | 0 | 0 | 0 | 0 | 0 | 0 | 0 | 0 | 0 | 0 | 0 | 0 | 0 | 1 |
| **6** | 0 | 0 | 0 | 0 | 0 | 0 | 0 | 0 | 0 | 0 | 0 | 0 | 0 | 0 | 0 | 1 |
| **7** | 0 | 0 | 0 | 0 | 0 | 0 | 0 | 0 | 0 | 0 | 0 | 0 | 0 | 0 | 0 | 1 |
| **8** | 0 | 0 | 0 | 0 | 0 | 0 | 0 | 0 | 0 | 0 | 0 | 0 | 0 | 0 | 0 | 1 |
| **9** | 0 | 0 | 0 | 0 | 0 | 0 | 0 | 0 | 0 | 0 | 0 | 0 | 0 | 0 | 0 | 1 |
| **11** | 0 | 0 | 0 | 0 | 0 | 0 | 0 | 0 | 0 | 0 | 0 | 0 | 0 | 0 | 0 | 1 |
| **12** | 0 | 0 | 0 | 0 | 0 | 0 | 0 | 0 | 0 | 0 | 0 | 0 | 0 | 0 | 0 | 1 |
| **13** | 0 | 0 | 0 | 0 | 0 | 0 | 0 | 0 | 0 | 0 | 0 | 0 | 0 | 0 | 0 | 1 |
| **14** | 0 | 0 | 0 | 0 | 0 | 0 | 0 | 0 | 0 | 0 | 0 | 0 | 0 | 0 | 0 | 1 |
| **16** | 0 | 0 | 0 | 0 | 0,004766 | 0,000636 | 0 | 0 | 0 | 0 | 0 | 0,010168 | 0 | 0 | 0,000636 | 0,983794 |
| **17** | 0 | 0,000289 | 0 | 0 | 0,001235 | 0,005381 | 2,30E-05 | 0 | 0 | 0 | 0,000208 | 0,000312 | 0 | 0 | 0 | 0,992552 |
| **18** | 0 | 0,000714 | 9,00E-06 | 1,70E-05 | 0,001239 | 0,001093 | 0 | 0 | 0 | 0 | 0,000439 | 0,000404 | 0 | 0 | 0 | 0,996086 |
| **20** | 0,000107 | 0,00075 | 0 | 0 | 0,000536 | 0,001929 | 0 | 0 | 0 | 0 | 0 | 0,00225 | 0 | 0 | 0 | 0,994428 |
| **21** | 0 | 0,000454 | 0 | 0 | 0,001362 | 0,000681 | 0 | 0 | 0 | 0 | 0 | 0 | 0 | 0 | 0 | 0,997503 |
| **22** | 0 | 0 | 0 | 0 | 0 | 0 | 0 | 0 | 0 | 0 | 0 | 0 | 0 | 0 | 0 | 1 |
| **23** | 0 | 0 | 0 | 0 | 0 | 0 | 0 | 0 | 0 | 0 | 0 | 0 | 0 | 0 | 0 | 1 |
| **24** | 0 | 0,001161 | 0 | 0 | 0,001347 | 0,001254 | 0 | 0 | 0 | 0 | 0,001114 | 4,60E-05 | 0 | 0 | 0 | 0,995078 |
| **25** | 0,027213 | 0,02567 | 0,022444 | 0,000421 | 0 | 0,002946 | 0,00014 | 0 | 0 | 0 | 0,000701 | 0,00014 | 0,023145 | 0,006453 | 0,005471 | 0,885257 |
| **26** | 0 | 0,001138 | 0 | 0 | 0 | 0 | 0 | 0 | 0 | 0 | 0 | 0 | 0 | 0 | 0 | 0,998862 |
| **27** | 0 | 0 | 0 | 0 | 0,159427 | 0 | 0 | 0 | 0 | 0 | 0 | 0 | 0,002311 | 0 | 0 | 0,838262 |
| **28** | 0 | 0 | 0 | 0 | 0 | 0 | 0,022989 | 0 | 0 | 0 | 0 | 0 | 0 | 0 | 0 | 0,977011 |
| **29** | 0 | 0 | 0 | 0 | 0 | 0 | 0 | 0 | 0 | 0 | 0 | 0 | 0 | 0 | 0 | 1 |
| **30** | 0 | 0,015219 | 0,000231 | 0 | 0,023289 | 0,001384 | 0 | 0 | 0 | 0 | 0,000692 | 0,000231 | 0 | 0 | 0,000461 | 0,958495 |
| **31** | 0 | 0 | 0 | 0 | 0 | 0 | 0 | 0 | 0 | 0 | 0 | 0 | 0 | 0 | 0 | 1 |
