# Supplementary material for: An agent-based model to simulate the transmission dynamics of bloodborne pathogens within hospitals
Source: PLoS Comput Biol. 2025 Feb 24;21(2):e1012850. doi: 10.1371/journal.pcbi.1012850 (PMC11882061; doi:10.1371/journal.pcbi.1012850)
Supplement: S6 Table — Each cell provides the probability of undergoing procedure j (in column) in ward I (in line) at each time-step. (DOCX) [file pcbi.1012850.s006.docx]

**Table S6.** Ward-specific probabilities of undergoing each of the procedures in the internal medicine department. Each cell provides the probability of undergoing procedure j (in column) in ward I (in line) at each time-step.

|  | **surgery** | **intravenous** | **sutures** | **blood_transfusion** | **blood_sample** | **injection** | **endoscopy** | **gastric_lavage** | **cardiac_catheter** | **dialysis** | **wound_dressing** | **bloodglucose** | **endotrachealintu** | **drainagecatheter** | **other_invproc** | **no_proc** |
| --- | --- | --- | --- | --- | --- | --- | --- | --- | --- | --- | --- | --- | --- | --- | --- | --- |
| **1** | 0 | 0,000671 | 0 | 0,000307 | 0,002989 | 0,000838 | 5,60E-05 | 8,40E-05 | 0 | 0 | 0 | 0,00109 | 0 | 0 | 0,000643 | 0,993323 |
| **2** | 0 | 0,000825 | 0 | 0,000474 | 0,003319 | 0,000577 | 0 | 0 | 0 | 0 | 0 | 0,000495 | 0 | 0 | 0,000124 | 0,994186 |
| **3** | 0 | 0,000814 | 0 | 0,000352 | 0,003629 | 0,001342 | 0 | 0 | 0 | 0 | 0 | 0,001254 | 0 | 0 | 4,40E-05 | 0,992565 |
| **4** | 0 | 0,001027 | 0 | 0,000486 | 0,003404 | 0,001243 | 0 | 0 | 0 | 0 | 0 | 0 | 0 | 0 | 0,000216 | 0,993625 |
| **5** | 0 | 0,001335 | 0 | 0,000103 | 0,003593 | 0,001386 | 0 | 0 | 0 | 0 | 0 | 0,001437 | 0 | 0 | 0 | 0,992147 |
| **6** | 0 | 0,000338 | 0 | 0,000169 | 0,002618 | 0,001267 | 0 | 0 | 0 | 0 | 0 | 0,0038 | 0 | 0 | 0,000507 | 0,991301 |
| **7** | 0 | 0,000792 | 0 | 0 | 0,003961 | 0,000226 | 0 | 0 | 0 | 0 | 0 | 0,000622 | 0 | 0 | 0,00017 | 0,994228 |
| **8** | 0 | 0,000839 | 5,20E-05 | 0,000446 | 0,002937 | 0,002727 | 0 | 0 | 2,60E-05 | 0,001049 | 0 | 0,001914 | 0 | 2,60E-05 | 0,000367 | 0,989615 |
| **9** | 0 | 0,000525 | 8,70E-05 | 0 | 0,002798 | 0,006557 | 0 | 0 | 0,000525 | 0 | 0 | 0,003934 | 0 | 0 | 0,000437 | 0,985137 |
| **11** | 0 | 0,001422 | 0 | 0 | 0,002133 | 0,003981 | 0,000142 | 0 | 0 | 0 | 0 | 0,003981 | 0 | 0 | 0 | 0,98834 |
| **12** | 0 | 9,00E-04 | 0 | 5,00E-04 | 0,002699 | 0,002024 | 0 | 5,00E-05 | 0 | 0 | 0 | 0,001524 | 0 | 0 | 0,000175 | 0,992129 |
| **13** | 0 | 0 | 0 | 0 | 0 | 0 | 0 | 0 | 0 | 0 | 0 | 0 | 0 | 0 | 0 | 1 |
| **14** | 0,000425 | 0,000161 | 0 | 0 | 0,000769 | 0,001183 | 0 | 0 | 0 | 0 | 0 | 7,00E-04 | 3,40E-05 | 0 | 0 | 0,996728 |
| **16** | 0 | 0 | 0 | 0 | 0 | 0 | 0 | 0 | 0 | 0 | 0 | 0 | 0 | 0 | 0 | 1 |
| **17** | 0 | 0 | 0 | 0 | 0 | 0 | 0 | 0 | 0 | 0 | 0 | 0 | 0 | 0 | 0 | 1 |
| **18** | 0 | 0 | 0 | 0 | 0 | 0 | 0 | 0 | 0 | 0 | 0 | 0 | 0 | 0 | 0 | 1 |
| **20** | 0 | 0 | 0 | 0 | 0 | 0 | 0 | 0 | 0 | 0 | 0 | 0 | 0 | 0 | 0 | 1 |
| **21** | 0 | 0 | 0 | 0 | 0 | 0 | 0 | 0 | 0 | 0 | 0 | 0 | 0 | 0 | 0 | 1 |
| **22** | 0 | 0 | 0 | 0 | 0 | 0 | 0 | 0 | 0 | 0 | 0 | 0 | 0 | 0 | 0 | 1 |
| **23** | 0 | 0 | 0 | 0 | 0 | 0 | 0 | 0 | 0 | 0 | 0 | 0 | 0 | 0 | 0 | 1 |
| **24** | 0 | 0 | 0 | 0 | 0 | 0 | 0 | 0 | 0 | 0 | 0 | 0 | 0 | 0 | 0 | 1 |
| **25** | 0,027778 | 0 | 0 | 0,013889 | 0 | 0,027778 | 0 | 0 | 0 | 0 | 0 | 0 | 0 | 0 | 0 | 0,930556 |
| **26** | 0,001339 | 0,001071 | 0 | 0,000268 | 0 | 0,00241 | 0,000268 | 0 | 0 | 0 | 0 | 0 | 0 | 0 | 0,004016 | 0,990628 |
| **27** | 0 | 0 | 0 | 0 | 0 | 0 | 0 | 0 | 0 | 0 | 0 | 0 | 0 | 0 | 0 | 1 |
| **28** | 0 | 0,002915 | 0 | 0 | 0,002915 | 0,002915 | 0,052478 | 0,002915 | 0 | 0 | 0 | 0 | 0 | 0 | 0 | 0,93586 |
| **29** | 0 | 0,000314 | 0 | 0 | 0,001572 | 0,000314 | 0 | 0 | 0 | 0 | 0 | 0 | 0 | 0 | 0 | 0,997799 |
| **30** | 0,000106 | 0,012239 | 0 | 0,001266 | 0,014771 | 0,001794 | 0 | 0,001372 | 0 | 0 | 0 | 0,003798 | 0 | 0 | 0,001583 | 0,963072 |
| **31** | 0 | 0,000502 | 1,00E-04 | 0,001305 | 0,003213 | 0,00241 | 0 | 0,001305 | 0 | 1,00E-04 | 0,001104 | 0,011245 | 0,000502 | 0 | 0,001506 | 0,976707 |
