# Supplementary material for: An agent-based model to simulate the transmission dynamics of bloodborne pathogens within hospitals
Source: PLoS Comput Biol. 2025 Feb 24;21(2):e1012850. doi: 10.1371/journal.pcbi.1012850 (PMC11882061; doi:10.1371/journal.pcbi.1012850)
Supplement: S7 Table — (DOCX) [file pcbi.1012850.s007.docx]

**Table S7.** Yearly initial quantity of new devices in each ward for the high-resource setting

| **Ward**  **Device** | **1** | **2** | **3** | **4** | **5** | **6** | **7** | **8** | **9** | **11** | **12** | **13** | **14** | **16** | **17** | **18** | **20** | **21** | **22** | **23** | **24** | **25** | **26** | **27** | **28** | **29** | **30** | **31** |
| --- | --- | --- | --- | --- | --- | --- | --- | --- | --- | --- | --- | --- | --- | --- | --- | --- | --- | --- | --- | --- | --- | --- | --- | --- | --- | --- | --- | --- |
| **Syringes** | 1155263 | 1654356 | 1984501 | 708925 | 923282 | 484672 | 577928 | 1884121 | 880828 | 397425 | 1596235 | 0 | 1775710 | 81079 | 2858066 | 1346126 | 112612 | 46574 | 0 | 0 | 266490 | 1012436 | 119147 | 357612 | 306 | 48462 | 39879 | 419600 |
| **IV Set** | 21171 | 40436 | 33712 | 15993 | 19258 | 4664 | 7657 | 56117 | 7030 | 6760 | 33219 | 0 | 33252 | 0 | 58692 | 205191 | 17417 | 5204 | 0 | 0 | 59330 | 845891 | 11308 | 0 | 162 | 576 | 30441 | 10147 |
| **IV Cannula** | 13550 | 25878 | 21571 | 10237 | 12327 | 2984 | 4900 | 35914 | 4498 | 4325 | 21261 | 0 | 21278 | 0 | 28756 | 100547 | 8537 | 2548 | 0 | 0 | 29069 | 415002 | 6613 | 0 | 162 | 371 | 30441 | 6497 |
| **Scalpel** | 0 | 0 | 0 | 0 | 0 | 0 | 0 | 0 | 0 | 0 | 0 | 0 | 17263880 | 0 | 0 | 0 | 55 | 0 | 0 | 0 | 0 | 39434 | 2149057 | 0 | 0 | 0 | 144 | 0 |
| **Lancet** | 15249 | 9251 | 23820 | 0 | 12718 | 22324 | 3974 | 30616 | 17948 | 12712 | 24539 | 0 | 23393 | 685564 | 615017 | 1163877 | 449300 | 0 | 0 | 0 | 20551 | 25776 | 0 | 0 | 0 | 0 | 6046 | 39707 |
| **Surgical needles & suture kits** | 604358 | 813480 | 810317 | 312197 | 369568 | 214978 | 316054 | 619479 | 179875 | 78270 | 546694 | 0 | 504489 | 20409490 | 159796014 | 237981903 | 7371332 | 7761866 | 0 | 0 | 62398459 | 444178020 | 93270 | 88643180 | 160 | 21811 | 39652 | 253788 |
| **Endotracheal tube** | 0 | 0 | 0 | 0 | 0 | 0 | 0 | 0 | 0 | 0 | 0 | 0 | 13073 | 0 | 0 | 0 | 0 | 0 | 0 | 0 | 0 | 10756 | 0 | 64 | 0 | 0 | 0 | 21369 |
| **Drainage catheter** | 0 | 0 | 0 | 0 | 0 | 0 | 0 | 145 | 0 | 0 | 0 | 0 | 0 | 0 | 0 | 0 | 0 | 0 | 0 | 0 | 0 | 6867 | 0 | 0 | 0 | 0 | 0 | 0 |
| **Gastric lavage tube** | 405 | 0 | 0 | 0 | 0 | 0 | 0 | 0 | 0 | 0 | 334 | 0 | 0 | 0 | 0 | 0 | 0 | 0 | 0 | 0 | 0 | 0 | 0 | 0 | 149 | 0 | 2086 | 2316 |
| **Endoscope** | 4096 | 0 | 0 | 0 | 0 | 0 | 0 | 0 | 0 | 2507 | 0 | 0 | 0 | 0 | 8396 | 0 | 0 | 0 | 0 | 0 | 0 | 4688 | 2289 | 0 | 2978 | 0 | 0 | 0 |
