# Supplementary material for: An agent-based model to simulate the transmission dynamics of bloodborne pathogens within hospitals
Source: PLoS Comput Biol. 2025 Feb 24;21(2):e1012850. doi: 10.1371/journal.pcbi.1012850 (PMC11882061; doi:10.1371/journal.pcbi.1012850)
Supplement: S9 Table — (DOCX) [file pcbi.1012850.s009.docx]

**Table S9.** Yearly initial quantity of new devices in each ward for the low-resource setting

| **Ward**  **Device** | **1** | **2** | **3** | **4** | **5** | **6** | **7** | **8** | **9** | **11** | **12** | **13** | **14** | **16** | **17** | **18** | **20** | **21** | **22** | **23** | **24** | **25** | **26** | **27** | **28** | **29** | **30** | **31** |
| --- | --- | --- | --- | --- | --- | --- | --- | --- | --- | --- | --- | --- | --- | --- | --- | --- | --- | --- | --- | --- | --- | --- | --- | --- | --- | --- | --- | --- |
| **Syringes** | 288816 | 413589 | 496125 | 177231 | 230821 | 121168 | 144482 | 471030 | 220207 | 99356 | 399059 | 0 | 443928 | 20270 | 714516 | 336531 | 28153 | 11644 | 0 | 0 | 66623 | 253109 | 29787 | 89403 | 76 | 12116 | 9970 | 104900 |
| **IV Set** | 5293 | 10109 | 8428 | 3998 | 4814 | 1166 | 1914 | 14029 | 1757 | 1690 | 8305 | 0 | 8313 | 0 | 14673 | 51298 | 4354 | 1301 | 0 | 0 | 14833 | 211473 | 2827 | 0 | 40 | 144 | 7610 | 2537 |
| **IV Cannula** | 3387 | 6470 | 5393 | 2559 | 3082 | 746 | 1225 | 8979 | 1124 | 1081 | 5315 | 0 | 5320 | 0 | 7189 | 25137 | 2134 | 637 | 0 | 0 | 7267 | 103751 | 1653 | 0 | 40 | 93 | 7610 | 1624 |
| **Scalpel** | 0 | 0 | 0 | 0 | 0 | 0 | 0 | 0 | 0 | 0 | 0 | 0 | 4315970 | 0 | 0 | 0 | 14 | 0 | 0 | 0 | 0 | 9859 | 537264 | 0 | 0 | 0 | 36 | 0 |
| **Lancet** | 3812 | 2313 | 5955 | 0 | 3180 | 5581 | 994 | 7654 | 4487 | 3178 | 6135 | 0 | 5848 | 171391 | 153754 | 290969 | 112325 | 0 | 0 | 0 | 5138 | 6444 | 0 | 0 | 0 | 0 | 1512 | 9927 |
| **Surgical needles & suture kits** | 151090 | 203370 | 202579 | 78049 | 92392 | 53744 | 79013 | 154870 | 44969 | 19567 | 136674 | 0 | 126122 | 5102373 | 39949004 | 59495476 | 1842833 | 1940466 | 0 | 0 | 15599615 | 111044505 | 23318 | 22160795 | 40 | 5453 | 9913 | 63447 |
| **Endotracheal tube** | 0 | 0 | 0 | 0 | 0 | 0 | 0 | 0 | 0 | 0 | 0 | 0 | 3268 | 0 | 0 | 0 | 0 | 0 | 0 | 0 | 0 | 2689 | 0 | 16 | 0 | 0 | 0 | 5342 |
| **Drainage catheter** | 0 | 0 | 0 | 0 | 0 | 0 | 0 | 36 | 0 | 0 | 0 | 0 | 0 | 0 | 0 | 0 | 0 | 0 | 0 | 0 | 0 | 1717 | 0 | 0 | 0 | 0 | 0 | 0 |
| **Gastric lavage tube** | 101 | 0 | 0 | 0 | 0 | 0 | 0 | 0 | 0 | 0 | 84 | 0 | 0 | 0 | 0 | 0 | 0 | 0 | 0 | 0 | 0 | 0 | 0 | 0 | 37 | 0 | 522 | 579 |
| **Endoscope** | 1024 | 0 | 0 | 0 | 0 | 0 | 0 | 0 | 0 | 627 | 0 | 0 | 0 | 0 | 2099 | 0 | 0 | 0 | 0 | 0 | 0 | 1172 | 572 | 0 | 744 | 0 | 0 | 0 |
