# Supplementary material for: An agent-based model to simulate the transmission dynamics of bloodborne pathogens within hospitals
Source: PLoS Comput Biol. 2025 Feb 24;21(2):e1012850. doi: 10.1371/journal.pcbi.1012850 (PMC11882061; doi:10.1371/journal.pcbi.1012850)
Supplement: S10 Table — (DOCX) [file pcbi.1012850.s010.docx]

**Table S10.** Yearly initial quantity of previously used devices in each ward for the low-resource setting

| **Ward**  **Device** | **1** | **2** | **3** | **4** | **5** | **6** | **7** | **8** | **9** | **11** | **12** | **13** | **14** | **16** | **17** | **18** | **20** | **21** | **22** | **23** | **24** | **25** | **26** | **27** | **28** | **29** | **30** | **31** |
| --- | --- | --- | --- | --- | --- | --- | --- | --- | --- | --- | --- | --- | --- | --- | --- | --- | --- | --- | --- | --- | --- | --- | --- | --- | --- | --- | --- | --- |
| **Syringes** | 0 | 0 | 0 | 0 | 0 | 0 | 0 | 0 | 0 | 0 | 0 | 0 | 0 | 0 | 0 | 0 | 0 | 0 | 0 | 0 | 0 | 0 | 0 | 0 | 76 | 0 | 9970 | 0 |
| **IV Set** | 188 | 0 | 0 | 386 | 0 | 0 | 278 | 63 | 122 | 0 | 463 | 0 | 0 | 0 | 0 | 0 | 0 | 0 | 0 | 0 | 0 | 0 | 0 | 0 | 40 | 12 | 7610 | 438 |
| **IV Cannula** | 997 | 1422 | 1246 | 948 | 426 | 5 | 528 | 2295 | 379 | 171 | 1699 | 0 | 1068 | 0 | 0 | 0 | 0 | 0 | 0 | 0 | 0 | 0 | 196 | 0 | 40 | 33 | 7610 | 756 |
| **Scalpel** | 0 | 0 | 0 | 0 | 0 | 0 | 0 | 0 | 0 | 0 | 0 | 0 | 0 | 0 | 0 | 0 | 29 | 0 | 0 | 0 | 0 | 6871 | 0 | 0 | 0 | 0 | 36 | 0 |
| **Lancet** | 1196 | 769 | 1365 | 0 | 416 | 198 | 419 | 1721 | 1292 | 418 | 1699 | 0 | 1986 | 0 | 0 | 0 | 0 | 0 | 0 | 0 | 0 | 0 | 0 | 0 | 0 | 0 | 1512 | 4457 |
| **Surgical needles & suture kits** | 0 | 0 | 0 | 0 | 0 | 0 | 0 | 0 | 0 | 0 | 0 | 0 | 0 | 0 | 0 | 0 | 0 | 0 | 0 | 0 | 0 | 0 | 0 | 0 | 40 | 0 | 9913 | 0 |
| **Endotracheal tube** | 0 | 0 | 0 | 0 | 0 | 0 | 0 | 0 | 0 | 0 | 0 | 0 | 0 | 0 | 0 | 0 | 0 | 0 | 0 | 0 | 0 | 5138 | 0 | 31 | 0 | 0 | 0 | 0 |
| **Drainage catheter** | 0 | 0 | 0 | 0 | 0 | 0 | 0 | 36 | 0 | 0 | 0 | 0 | 0 | 0 | 0 | 0 | 0 | 0 | 0 | 0 | 0 | 1717 | 0 | 0 | 0 | 0 | 0 | 0 |
| **Gastric lavage tube** | 101 | 0 | 0 | 0 | 0 | 0 | 0 | 0 | 0 | 0 | 84 | 0 | 0 | 0 | 0 | 0 | 0 | 0 | 0 | 0 | 0 | 0 | 0 | 0 | 37 | 0 | 522 | 579 |
| **Endoscope** | 0 | 0 | 0 | 0 | 0 | 0 | 0 | 0 | 0 | 0 | 0 | 0 | 0 | 0 | 0 | 0 | 0 | 0 | 0 | 0 | 0 | 0 | 0 | 0 | 744 | 0 | 0 | 0 |
