# Supplementary figures and images for: An agent-based model to simulate the transmission dynamics of bloodborne pathogens within hospitals
Source: PLoS Comput Biol. 2025 Feb 24;21(2):e1012850. doi: 10.1371/journal.pcbi.1012850 (PMC11882061; doi:10.1371/journal.pcbi.1012850)

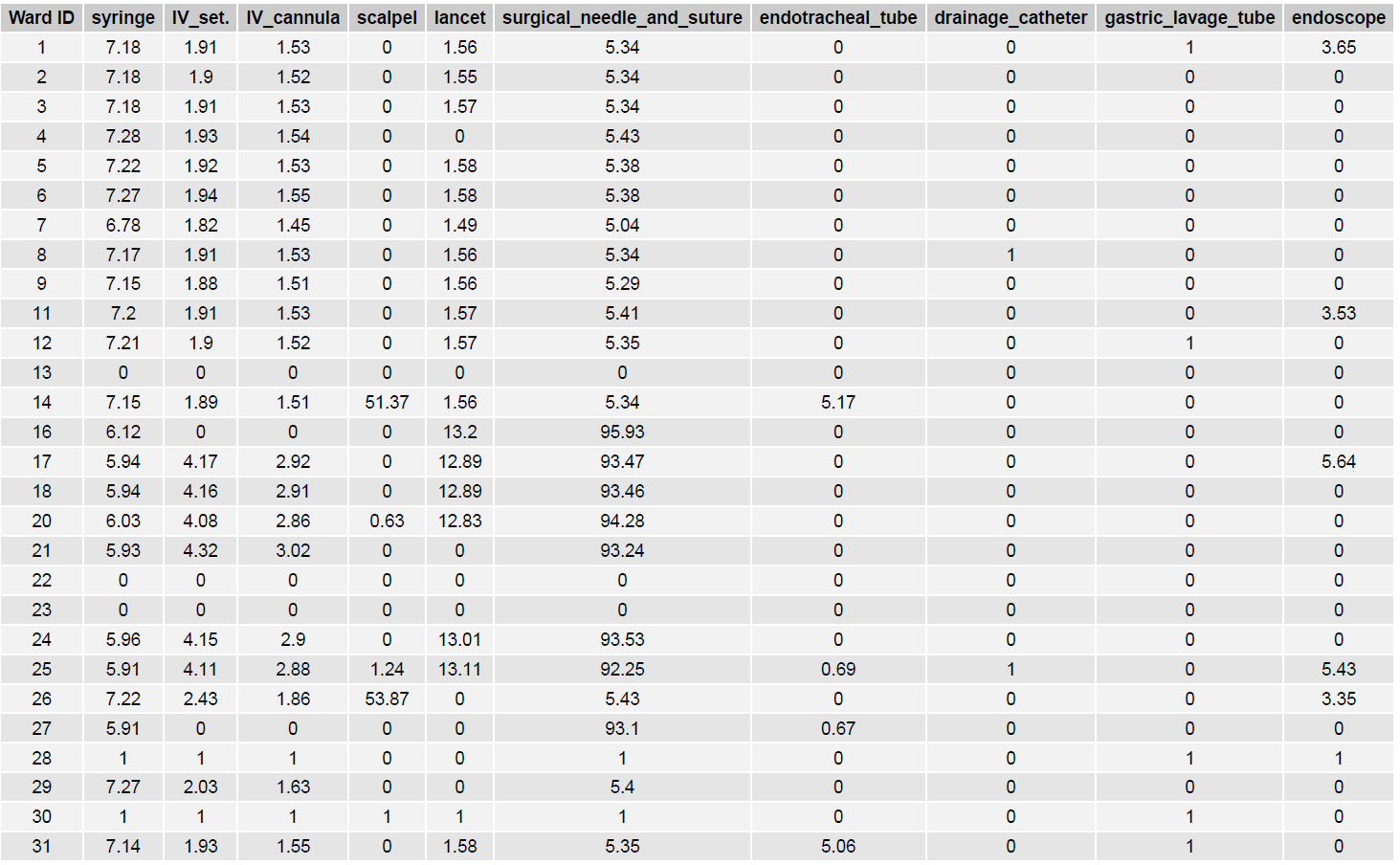

Supplement: S1 Fig — There are insufficient devices to cover the hospital’s needs when the value <1. (PNG) [file pcbi.1012850.s011.png]

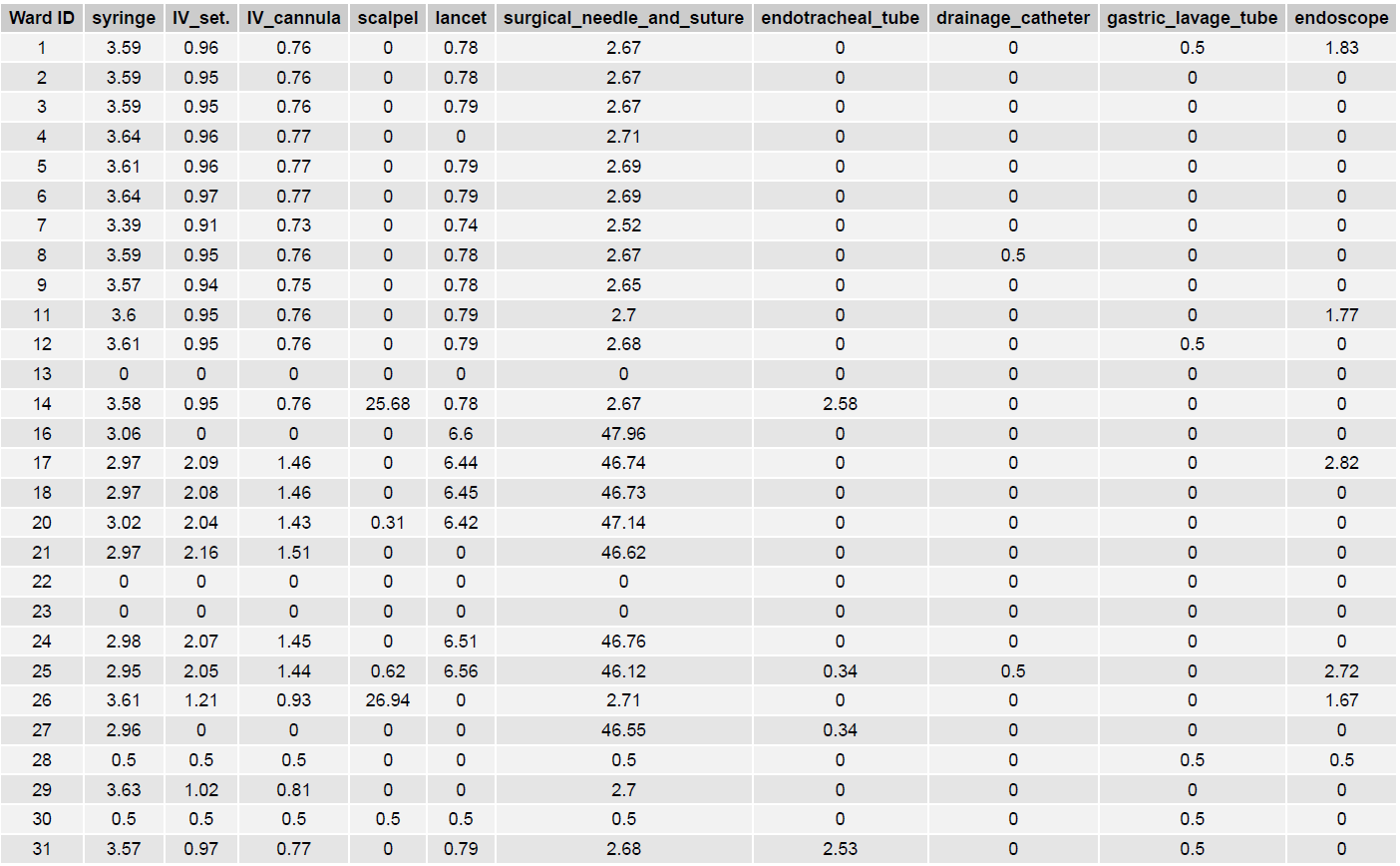

Supplement: S2 Fig — There are insufficient devices to cover the hospital’s needs when the value <1. (PNG) [file pcbi.1012850.s012.png]

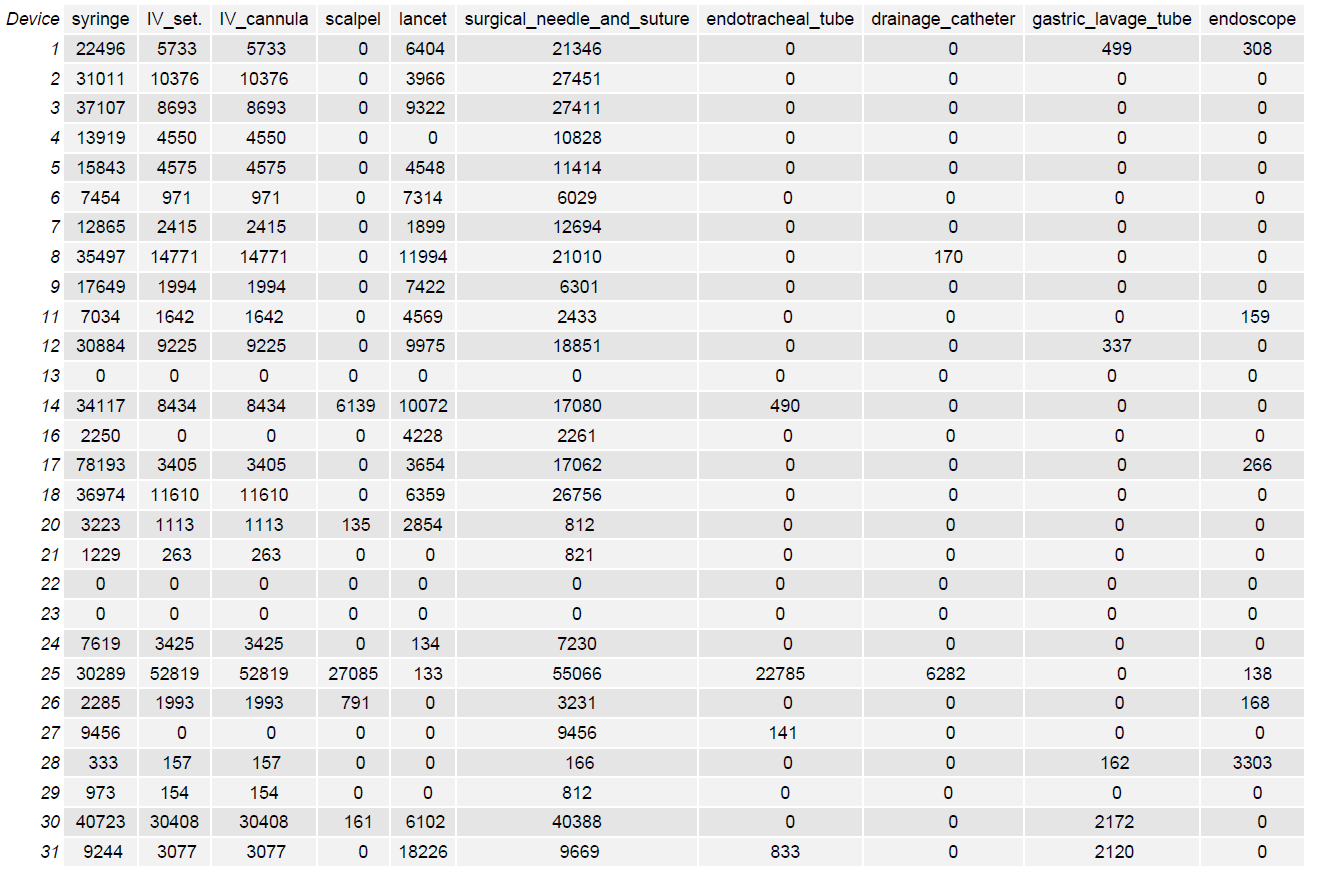

Supplement: S3 Fig — (PNG) [file pcbi.1012850.s013.png]

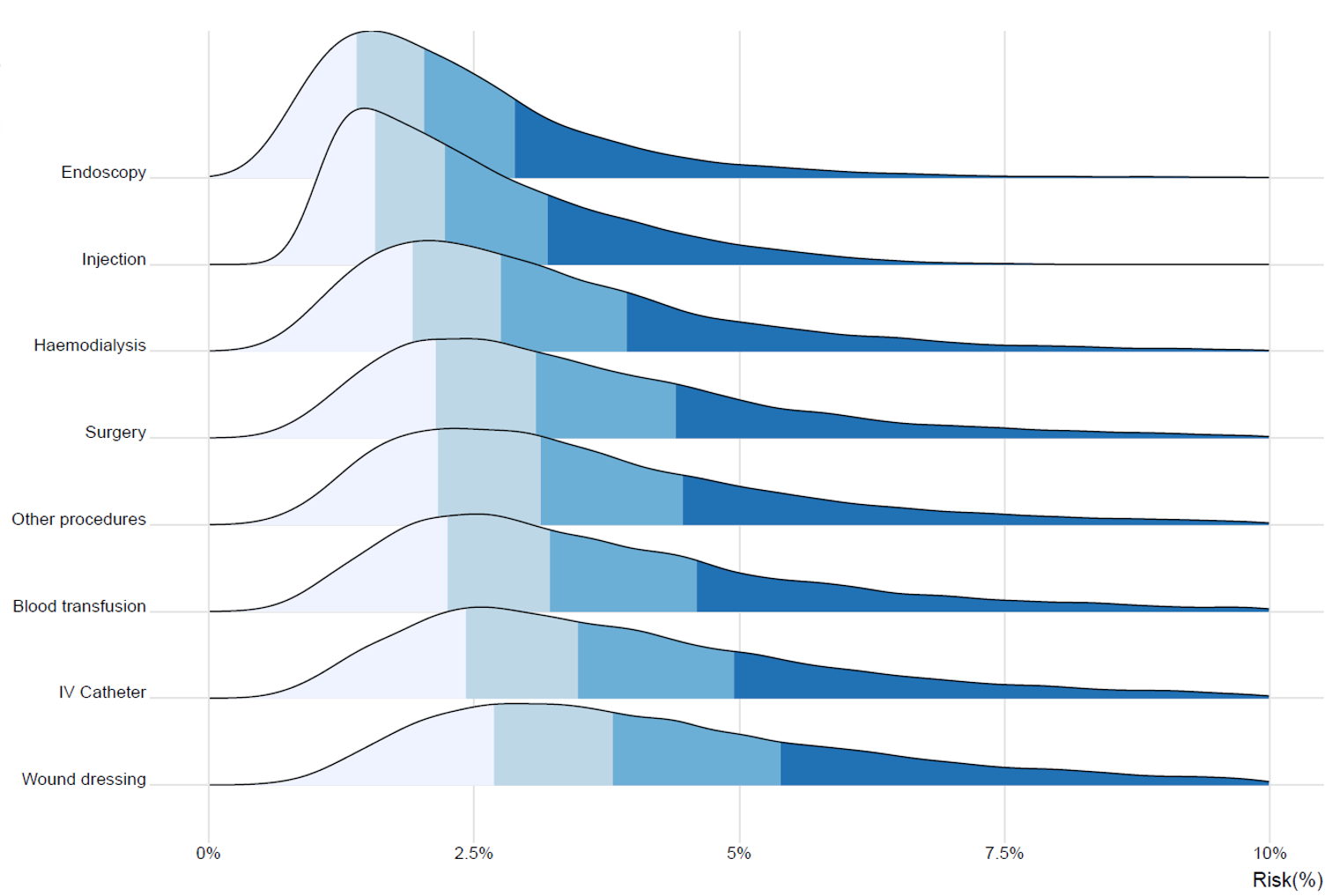

Supplement: S4 Fig — (PNG) [file pcbi.1012850.s014.png]

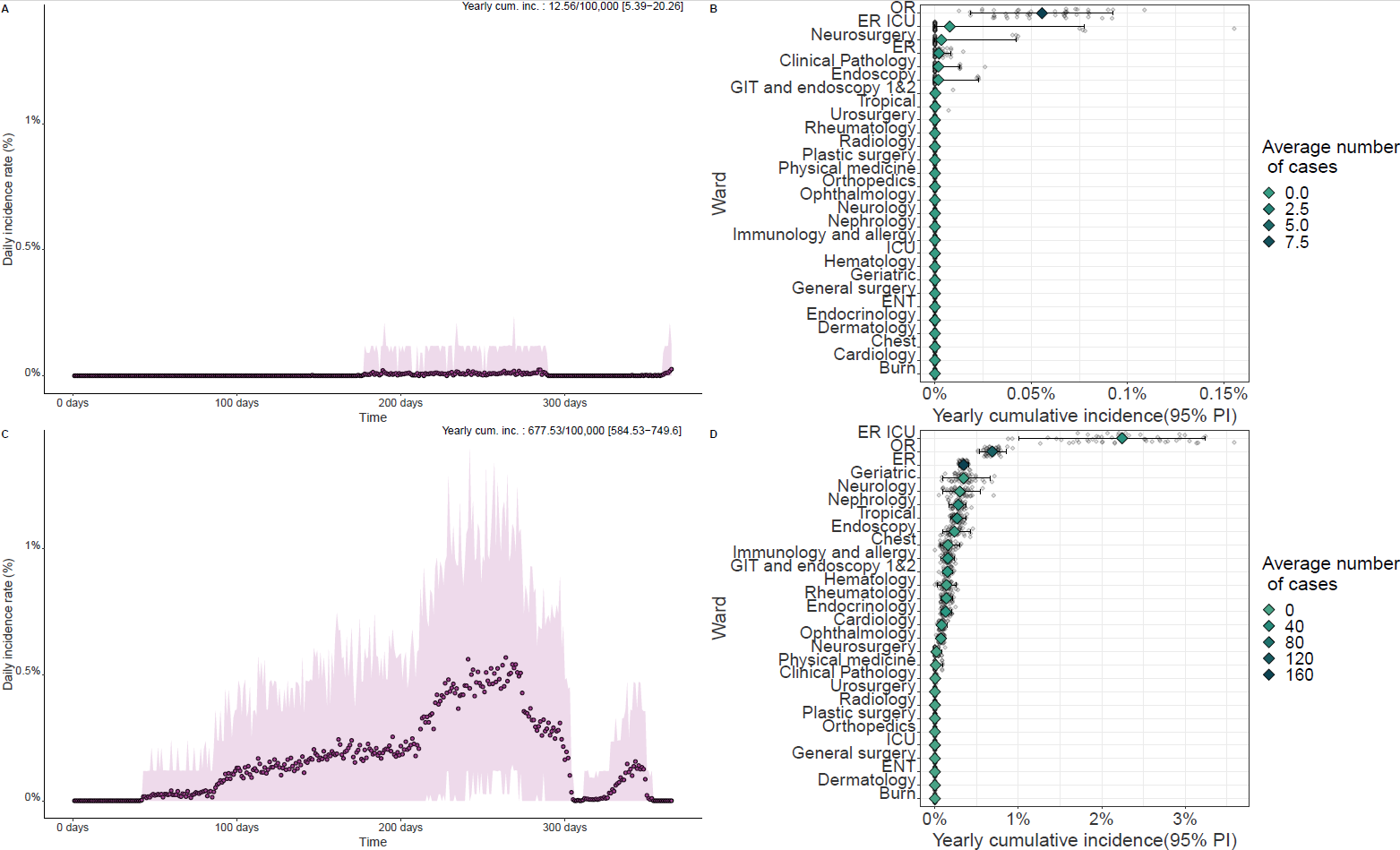

Supplement: S5 Fig — (A) and (B): Daily incidence rate for the high- and low-resource hospital, respectively. (C) and (D) Yearly cumulative incidence (mean and 95% PI) and average number of cases for each ward, ranked by mean cumulative incidence values. (PNG) [file pcbi.1012850.s015.png]

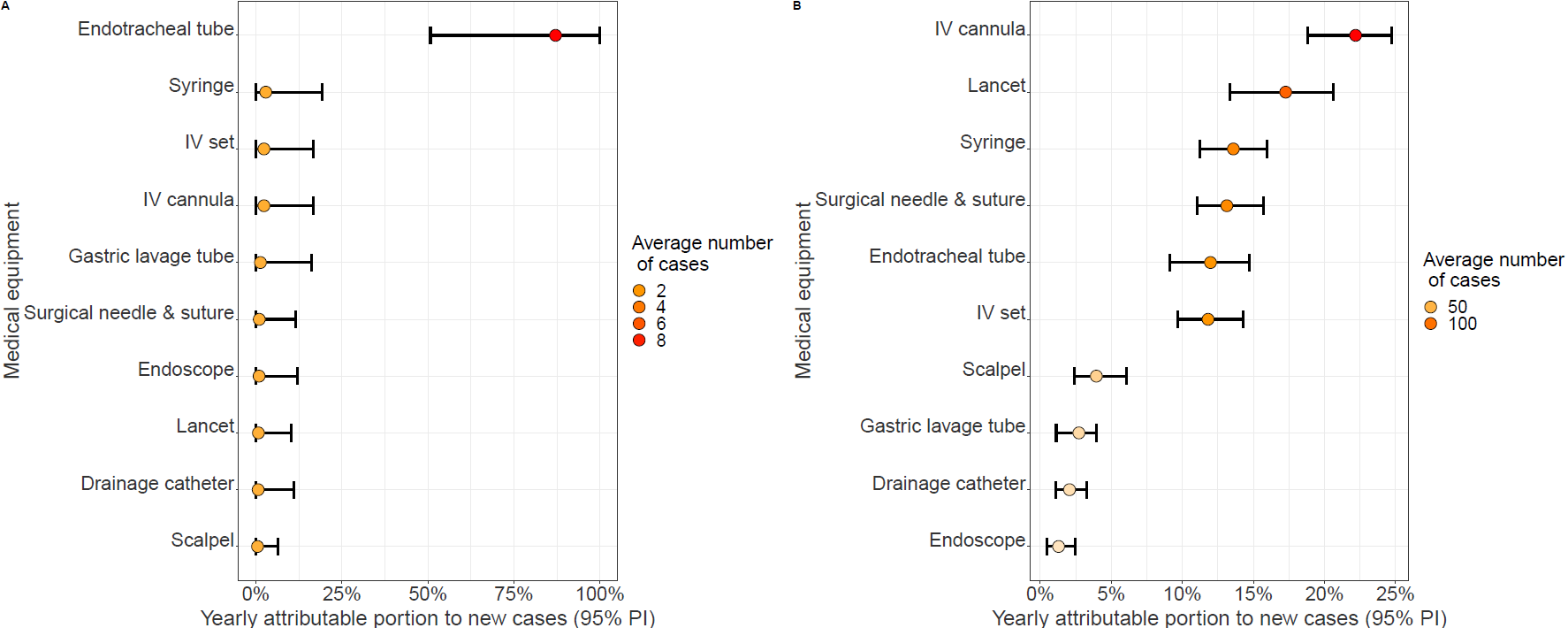

Supplement: S6 Fig — (PNG) [file pcbi.1012850.s016.png]

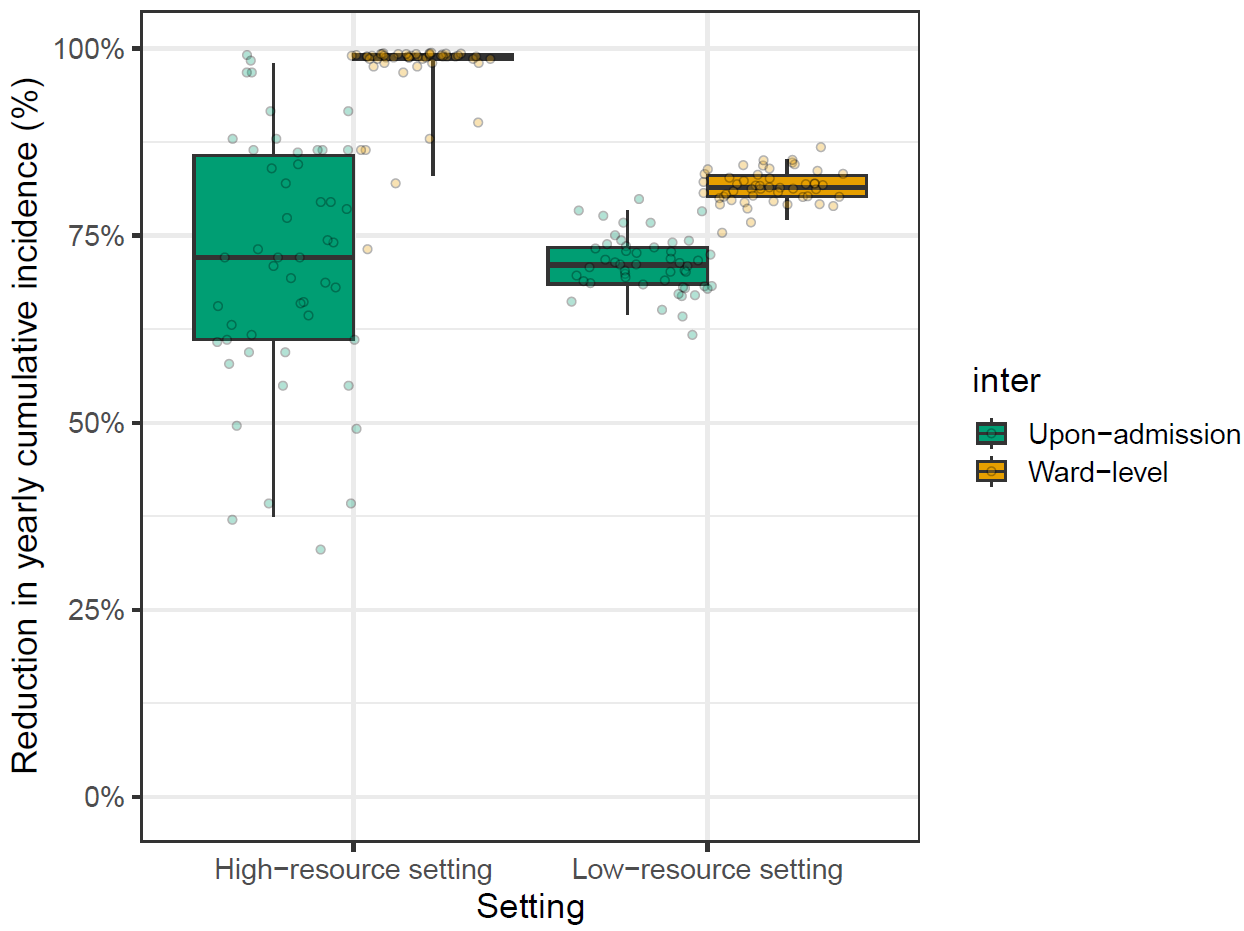

Supplement: S7 Fig — Baseline scenarios correspond to the no-intervention scenarios. In both high-resource and low-resource settings, 53,640 patients (72%) are screened (i) either systematically (in the three most at-risk wards) or (ii) randomly upon admission. (PNG) [file pcbi.1012850.s017.png]

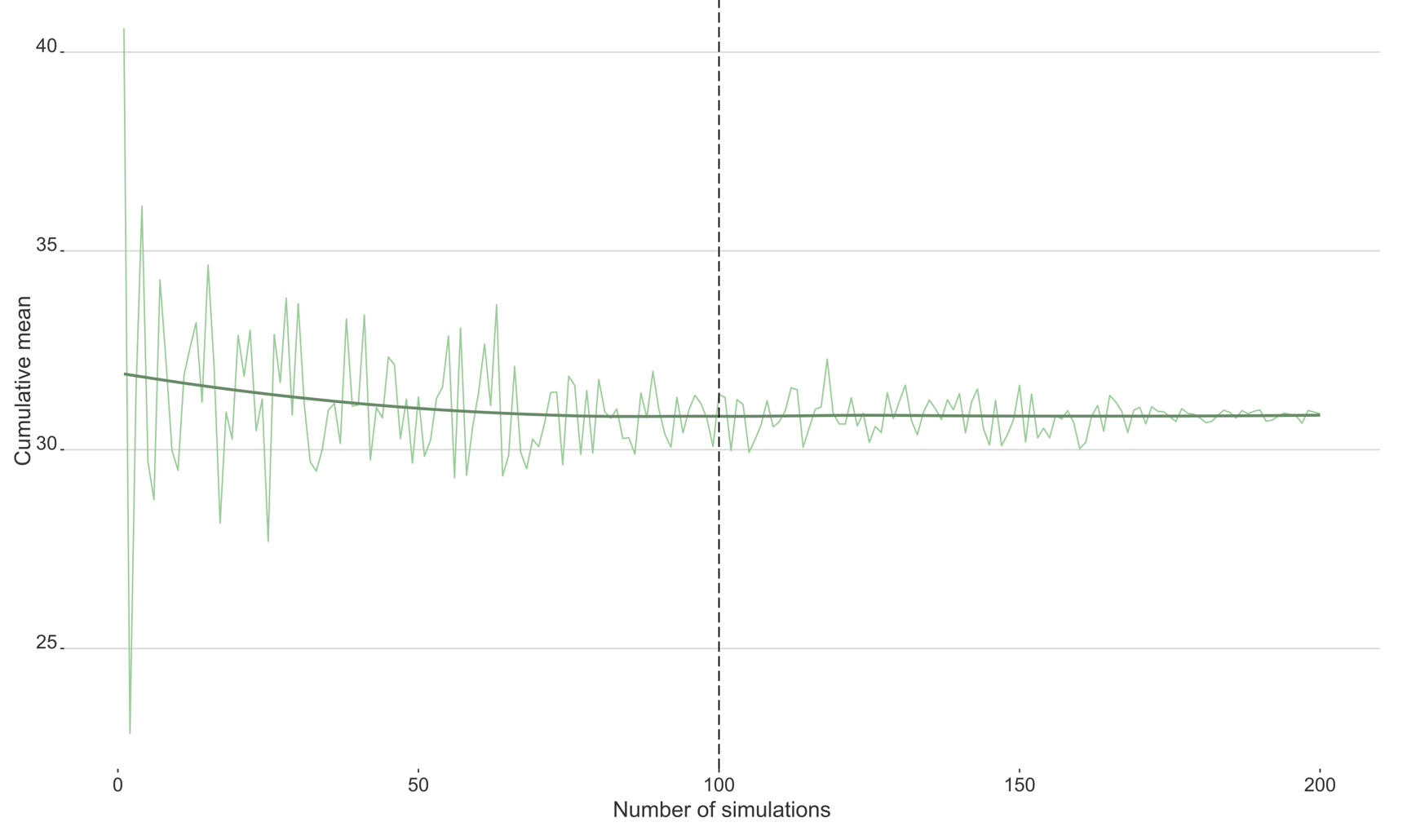

Supplement: S8 Fig — This graph shows that the mean seems to converge after 100 simulations. (PNG) [file pcbi.1012850.s018.png]

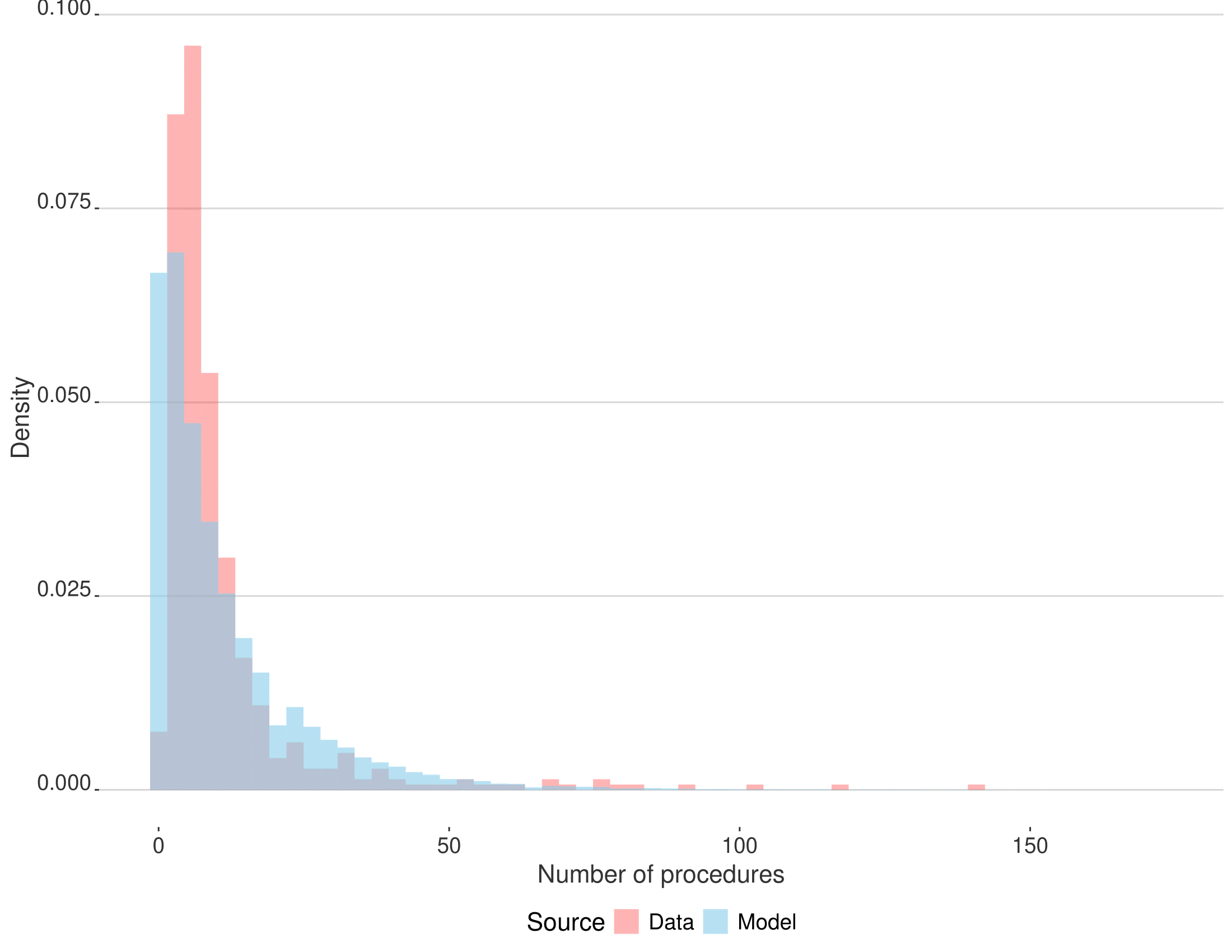

Supplement: S9 Fig — The average number of procedures extracted from the data was of 11.22 (95% CI [9.90–12.54]; median: 7) and was estimated at 11.36 (95% CI [11.26–11.46]; median: 7) using our model. (PNG) [file pcbi.1012850.s019.png]

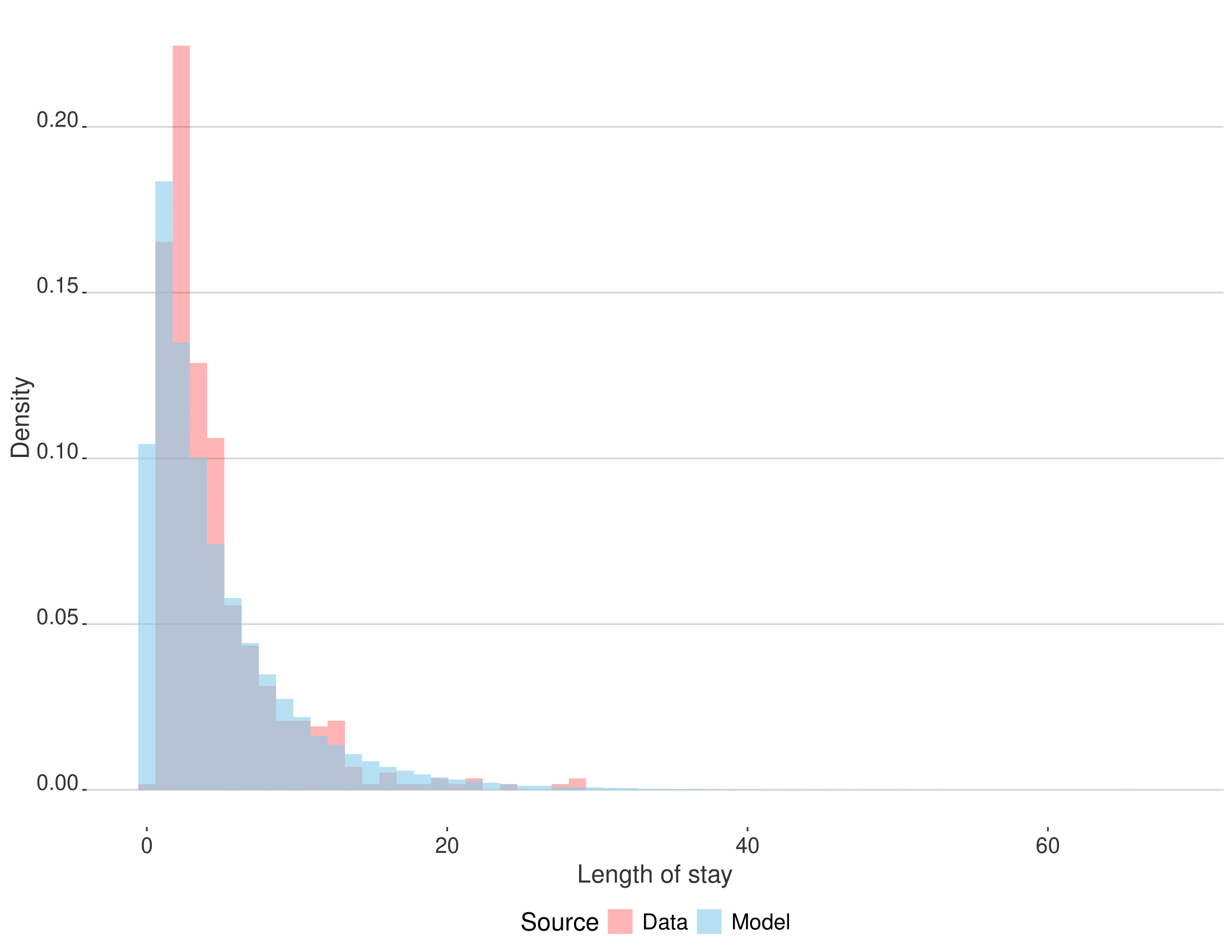

Supplement: S10 Fig — The average number of procedures extracted from the data was of 4.60 (95% CI [4.24–4.97]; median: 3.05) and was estimated at 4.68 (95% CI [4.65–4.72]; median: 3) using our model. (PNG) [file pcbi.1012850.s020.png]

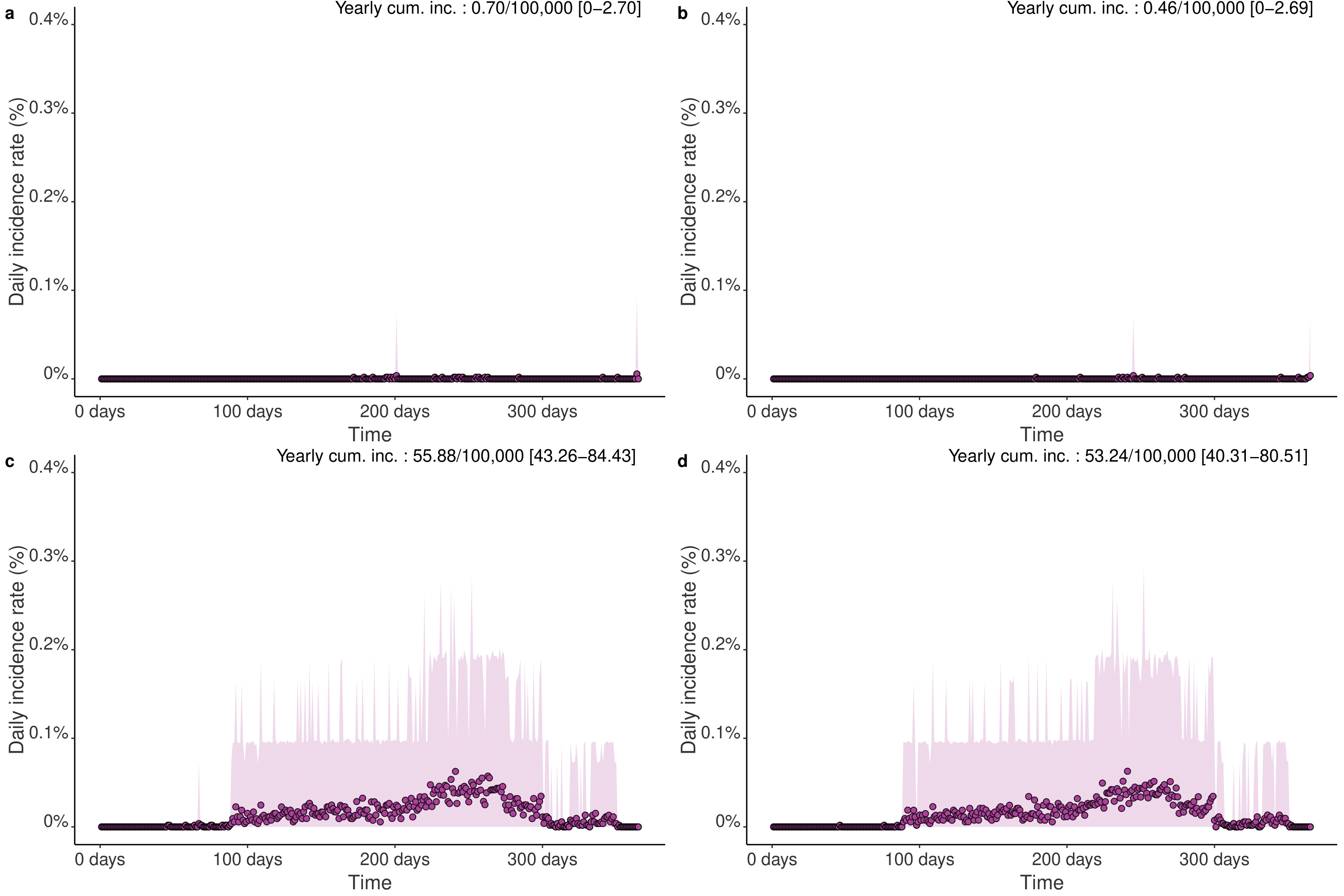

Supplement: S11 Fig — (PNG) [file pcbi.1012850.s021.png]

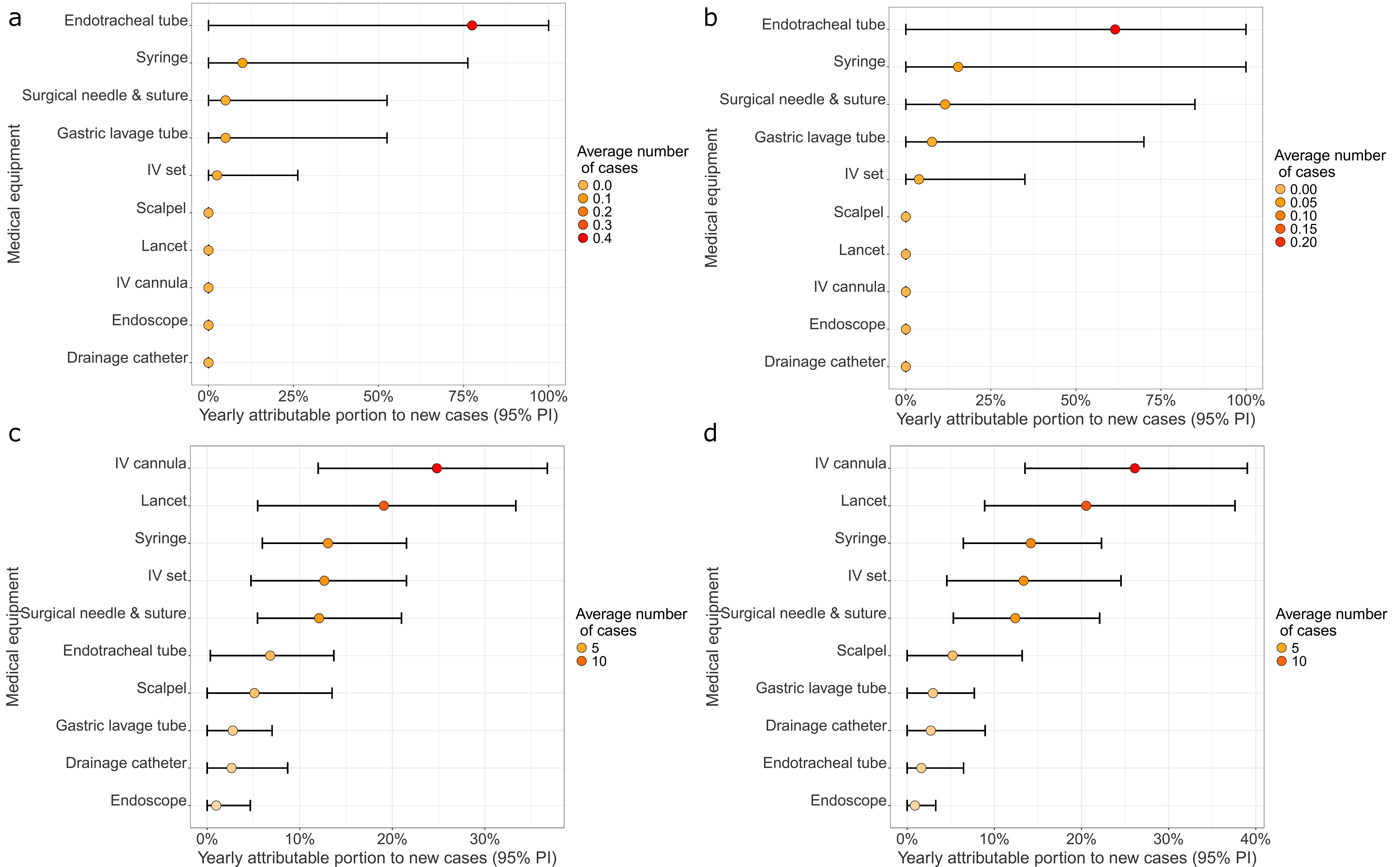

Supplement: S12 Fig — (PNG) [file pcbi.1012850.s022.png]

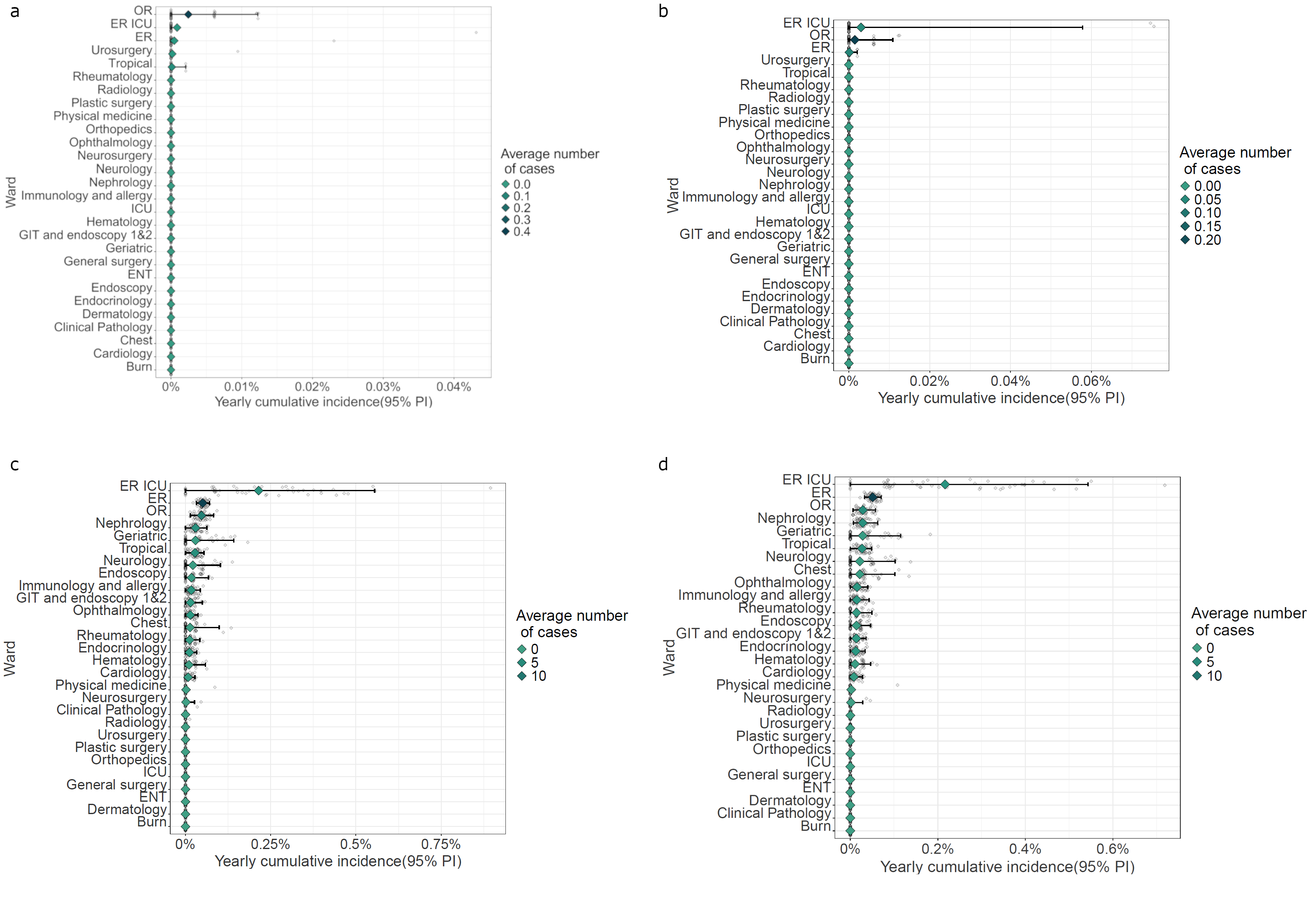

Supplement: S13 Fig — (PNG) [file pcbi.1012850.s023.png]
