# Supplementary material for: An agent-based model to simulate the transmission dynamics of bloodborne pathogens within hospitals
Source: PLoS Comput Biol. 2025 Feb 24;21(2):e1012850. doi: 10.1371/journal.pcbi.1012850 (PMC11882061; doi:10.1371/journal.pcbi.1012850)
Supplement: S1 Text — (DOCX) [file pcbi.1012850.s024.docx]

**Text S1.** Method used to estimate the resupply frequency

We consider here a device of which a quantity $Q_{tot}$ is bought by the hospital periodically at a given resupply frequency, and a quantity n per time step is required to perform procedures in the hospital. Informing $Q_{tot}$ by the yearly quantity of the device bought by the hospital, and denoting by tmax the total number of time steps over a year, we can then compute the ratio $r=Q_{tot}/(n\times t_{max})$ providing the average portion of needs fulfilled for this device.

In order to estimate the resupply frequency from observed data, we then describe device use over a 1-year period, from initial time t0 to final time tmax (see figure below, assuming that *r* < 1). At t0, we assume that the quantity of available supply of the device is $Q_{tot}\times r$. Starting from this initially available quantity, n devices are then used per time step until no new device is available at time tø. The available quantity of new devices then remains at zero, until device renewal at time $t_{\mu}$. Then again, n devices are used per time step until the end of the year.

We can then mathematically formulate the quantity of devices available at the end of the 1-year period as:

$$Q_{tot}-n\times(t_{max}-t_{\mu})$$

By assuming that the initial quantity of new devices for a given year is equal to the initial quantity of new devices for the next year, we can thus write that:

$$Q_{tot}\times r= Q_{tot}-n\times(t_{max}-t_{\mu})$$

From which we may compute the average number of time-steps $t_{\mu}$ between resupplies:

$$\boldsymbol{t}_{\boldsymbol{\mu}}\boldsymbol{=}t_{max}+\frac{\boldsymbol{Q}_{\boldsymbol{tot}}\boldsymbol{\times}\left( \boldsymbol{r-1} \right)}{\boldsymbol{n}}$$
