# Supplementary material for: An agent-based model to simulate the transmission dynamics of bloodborne pathogens within hospitals
Source: PLoS Comput Biol. 2025 Feb 24;21(2):e1012850. doi: 10.1371/journal.pcbi.1012850 (PMC11882061; doi:10.1371/journal.pcbi.1012850)
Supplement: Attachment — Submitted filename: Review_PCOMPBIOL-D-24-00422.docx [file pcbi.1012850.s025.docx]

Review

PCOMPBIOL-D-24-00422

It is stated that the main route of transmission of blood-borne pathogens in the community (HCV, HBV, HIV) occurs through the repetitive use of contaminated needles. However, exposure to contaminated body fluids (semen, vaginal fluid, blood ) through sexual relations is the most frequent route of transmission in the general population.

Within invasive procedures, devices have been included that are considered for single use and that have a solid recommendation for avoiding their reprocessing precisely because they are associated with outbreaks of transmission of blood pathogens, mainly needles and syringes, and intravenous equipment ( IV), lancets. Exposure to critical medical devices that in some centers with limited resources could be reprocessed and have a high risk has not been taken into account: central catheters and wire guides, hemodialysis catheters, intraosseous catheters, and bone marrow aspiration needles. Nor has exposure to surgical instruments been taken into account, except the scalpel, which are exposed to a large amount of blood. Although rare, transmission to patients after surgery and transmission from doctor to patient has been reported (https://www.cdc.gov/mmwr/preview/mmwrhtml/mm6407a1.htm, https:// doi.org/10.1016/j.athoracsur.2010.06.053, https://www.nejm.org/doi/full/10.1056/NEJM199602293340902,

Semi-critical medical devices (contact with mucosa but not with intravenous route) such as endotracheal tubes, gastric drains are frequently reprocessed due to limited resources. However, they can be safe for reuse if they have adequate cleaning, decontamination, disinfection, and sterilization. Since they are exposed to excretions and rarely to blood, they do not constitute a significant risk for transmission of blood pathogens but rather for possible transmission of pathogenic bacteria.

Within the model, effectiveness for all reprocessings of 80% is arbitrarily assumed, based on low adherence or resources. However, this needs to be carefully reviewed. It is argued that the effectiveness of "sterilization" was not found for each device. Therefore, an expert opinion was taken, taking up to 95% in the best conditions. However, there is literature on effectiveness in the reprocessing of endotracheal tubes (https://doi.org/10.1016/j.outlook.2022.08.004, https://doi.org/10.1016/j.jclinane.2007.02.009, https://doi.org/10.14238/pi51.2.2011.73-8,

The reuse of syringes and needles is a threat to patients' health. As I mentioned previously, outbreaks of blood pathogens have been reported due to reuse at the community and hospital level (https://pubmed.ncbi.nlm.nih.gov/20123446/). Although they may be able to be reprocessed in some centers, this is not an acceptable and very high-risk practice, regardless of the sterilization technique used.

There are several types of intravenous cannulas for peripheral use. They usually have a guide, a flexible part of plastic, or other flexible material to deliver intravenous fluids. These devices are for single use, and reprocessing is not feasible because the flexible part bends or breaks easily. If the device the model refers to is the central venous catheter, it must be corrected. Again, outbreaks of infections due to pathogenic microorganisms have been described due to the reuse and reprocessing of hemodialysis and intravenous catheters, making this practice not recommended.

In the discussion, we talk about reports of hepatitis B and C in Europe by Singh et al (ref 19), where although transmission to patients is evident, unspecified failures in prevention and control are identified, and transfusions as the main ones in units. of hemodialysis as the greatest risk. In the case of the CDC report (ref 20), 94% of the reports are from non-hospital settings. It is indicated that the model could predict these outbreaks, although the outbreaks report a small number of cases in many countries or states with heterogeneous characteristics.

I recommend reading literature on reprocessing and the use of single-use devices. https://www.who.int/publications/i/item/9789241549851, https://doi.org/10.1097/XEB.0000000000000146

I find your article very valuable and innovative. However, it is difficult to separate in practice the transmission of HCV, HBV, HIV through reprocessed medical devices from other bacterial and fungal hospital pathogens. Given that the reprocessing and reuse of devices is a frequent and heterogeneous practice in low-income countries, the model needs to be adjusted to include the specific risk for each device. I understand that the data from another publication by the same author has been used. However the categories of procedures are quite different

The model has found that the greatest attribution of transmission is due to the endotracheal tube, however, I find this result without biological plausibility. Although I have searched, I have not found a single reported case of any of the blood pathogens that have been attributed to using a reused endotracheal tube. However, they are identified as real risks in the guidelines. Although reprocessing and sterilizing endotracheal tubes may be common in low-resource settings, it is unlikely to go unreported.

Finally, it is very valuable that the model includes and supports screening as a strategy to prevent transmission. This model supports the performance of screening tests for blood pathogens in hospitalized individuals in different scenarios: preoperative, critical condition, before receiving a transfusion, and pregnancy. Although this may not be optimally feasible under low resource conditions, it is an important step to guide management decision-making.
