# Supplementary material for: An agent-based model to simulate the transmission dynamics of bloodborne pathogens within hospitals
Source: PLoS Comput Biol. 2025 Feb 24;21(2):e1012850. doi: 10.1371/journal.pcbi.1012850 (PMC11882061; doi:10.1371/journal.pcbi.1012850)
Supplement: Attachment — Submitted filename: Responses_to_reviewers_Plos_CB.docx [file pcbi.1012850.s026.docx]

**Response to reviewers**

We are very grateful to both reviewers for their comments. We believe that they have helped improve the clarity of the paper and reinforce our conclusions. In particular, we have added new paragraphs to the Discussion to provide more insight on our results’ implications. We have also clarified model equations and made our code available on GitHub (see the *Data Availability Statement*).

Following a specific comment from reviewer 1 which prompted us to further investigate the predicted key impact of endotracheal tubes in HCV infections in our simulations, we identified an error in our data treatment process. Indeed, in our initial analyses, endotracheal tubes were systematically associated with the “surgery” procedure, even though “endotracheal intubation” was also recorded as a specific procedure in the database. In our simulations, this led to endotracheal tubes being used twice in operations requiring intubation (around 70% of surgeries in our dataset) and once in operations not necessarily requiring intubation.

We have updated our results throughout the manuscript. Correcting this point led to a significant decrease in the predicted average number of endotracheal tubes used per year, from 57,357 to 23,831 (much closer to the annual available quantity of 22,500 in Ain Shams university hospital). As a consequence, although reuse of this type of device still occurred in our simulations, the predicted annual cumulative HCV infection incidence was lower, with an average of 1/100,000 in the high resource scenario (compared to 4/100,000 in our earlier results), and 60/100,000 in the low resource scenario (compared to 100/100,000). In addition, while endotracheal tubes remain the most at-risk device in the high-resource scenario, the most at-risk medical device in the low-resource scenario (Figure 3) has changed to intravenous cannula. However, our main messages regarding the ranking of the most at-risk wards on one hand, and the impact of potential interventions targeting high-risk wards for systematic screening on the other, remained unchanged.

Specific responses to both reviewers are detailed below.

**Reviewer 1**

- It is stated that the main route of transmission of blood-borne pathogens in the community (HCV, HBV, HIV) occurs through the repetitive use of contaminated needles. However, exposure to contaminated body fluids (semen, vaginal fluid, blood ) through sexual relations is the most frequent route of transmission in the general population.

**R :** We thank reviewer 1 for this remark. We initially wrote this sentence with HCV in mind, but we agree that the repetitive use of contaminated needles is not the main route of transmission of all the listed bloodborne pathogens. We changed the sentence to: “Injecting drug use is considered as one of the main routes of bloodborne pathogen diffusion in the community, but iatrogenic transmission may occur during invasive procedures when compliance with infection control measures is imperfect” (L 59-62)

- Within invasive procedures, devices have been included that are considered for single use and that have a solid recommendation for avoiding their reprocessing precisely because they are associated with outbreaks of transmission of blood pathogens, mainly needles and syringes, and intravenous equipment ( IV), lancets.

**R :** We agree that recommendations are against the reuse of these devices, but some publications show that there is still not a perfect adherence to these recommendations; even in high-income countries . We have added a sentence underlying this point in the discussion section: “Several of the devices mentioned in our results are intended to be only used once (23,24), but compliance with these recommendations is still not perfect, with contamination events still being reported, even in high-income countries (25, 26).” (L570-572)

- Exposure to critical medical devices that in some centres with limited resources could be reprocessed and have a high risk has not been taken into account: central catheters and wire guides, haemodialysis catheters, intraosseous catheters, and bone marrow aspiration needles.

**R:** We thank the reviewer for this useful remark. These medical devices were not included in our analyses because we did not have, in the IMMHOTHep database, any data on their use or availability. However, our model could easily include these devices if we had the necessary data. This limitation is now also underlined in the discussion section: “Moreover, we did not have access to data for every type of medical devices. In particular, high-risk devices such as intraosseous catheters or bone marrow aspiration needles were not taken into account.” (L544-546)

- Nor has exposure to surgical instruments been taken into account, except the scalpel, which are exposed to a large amount of blood. Although rare, transmission to patients after surgery and transmission from doctor to patient has been reported.

**R:** Again, we did not have data on the use or availability of other surgical devices. However, we had information on surgical procedures, which did take into account all the associated sub-procedures and were indeed assumed to be associated with a risk of HCV acquisition.

- Semi-critical medical devices (contact with mucosa but not with intravenous route) such as endotracheal tubes, gastric drains are frequently reprocessed due to limited resources. However, they can be safe for reuse if they have adequate cleaning, decontamination, disinfection, and sterilization. Since they are exposed to excretions and rarely to blood, they do not constitute a significant risk for transmission of blood pathogens but rather for possible transmission of pathogenic bacteria.

**R:** We agree that these devices are frequently reprocessed and should in theory be adequately decontaminated and sterilized before reuse. However, if disinfection practices are imperfect (which is taken into account in our model with the probability of effective disinfection), there might still be a risk of pathogen transmission, including of bloodborne pathogens. In particular, our previous meta-analysis published in 2022 (<https://onlinelibrary.wiley.com/doi/full/10.1111/apt.17106>) suggests that a history of endoscopy is associated with a significant risk of HCV positivity in countries with an HCV prevalence above 5%.

- Within the model, effectiveness for all reprocessings of 80% is arbitrarily assumed, based on low adherence or resources. However, this needs to be carefully reviewed. It is argued that the effectiveness of "sterilization" was not found for each device. Therefore, an expert opinion was taken, taking up to 95% in the best conditions. However, there is literature on effectiveness in the reprocessing of endotracheal tubes (https://doi.org/10.1016/j.outlook.2022.08.004, https://doi.org/10.1016/j.jclinane.2007.02.009, <https://doi.org/10.14238/pi51.2.2011.73-8>,

**R:** We thank the reviewer for these interesting references. The provided references demonstrate the potential effectiveness of disinfection of endotracheal tubes in perfectly controlled cases; however, we can expect that these recommendations are not always perfectly followed. Although the first two references show that different regimens of disinfection can stop bacterial growth, the results of the third reference provided (Elisa *et al.*), found a disinfection efficiency of 71% to 83% (the authors still observed growth of some bacterial species after disinfection). The results are consistent with our second scenario, which assumes an efficient disinfection probability of 80%. We have added this reference to the Discussion section:

“However, the probability of efficient disinfection of 80% considered for endotracheal tubes in the low-resource setting scenario seems to be in line with a study by Elisa *et al.* (20) showing disinfection efficiencies of 71% and 83%.” (L548-550)

- The reuse of syringes and needles is a threat to patients' health. As I mentioned previously, outbreaks of blood pathogens have been reported due to reuse at the community and hospital level (https://pubmed.ncbi.nlm.nih.gov/20123446/). Although they may be able to be reprocessed in some centers, this is not an acceptable and very high-risk practice, regardless of the sterilization technique used.

**R:** We fully agree with reviewer 1 that these practices should not be implemented and thank him for the reference provided, which we now cite in the introduction. The aim of our work was precisely to assess the impact of reusing this type of device when sterile devices are not available, and illustrate potential associated risks, rather than encouraging the reuse of such devices.

- There are several types of intravenous cannulas for peripheral use. They usually have a guide, a flexible part of plastic, or other flexible material to deliver intravenous fluids. These devices are for single use, and reprocessing is not feasible because the flexible part bends or breaks easily. If the device the model refers to is the central venous catheter, it must be corrected. Again, outbreaks of infections due to pathogenic microorganisms have been described due to the reuse and reprocessing of hemodialysis and intravenous catheters, making this practice not recommended.

**R:** We agree with the reviewer that differentiating between CVCs and other types of catheters is important. However, within the IMMHOTHep database, the available data refers to intravenous catheters in general, without specification on the type of catheter, leading us to assume that all “catheters” could be reused, in a worst-case scenario. Again, we fully agree that catheters should not be reused, and the aim of our work was to illustrate the risks associated with reusing this type of device when sterile devices are not available.

- In the discussion, we talk about reports of hepatitis B and C in Europe by Singh et al (ref 19), where although transmission to patients is evident, unspecified failures in prevention and control are identified, and transfusions as the main ones in units. of hemodialysis as the greatest risk. In the case of the CDC report (ref 20), 94% of the reports are from non-hospital settings. It is indicated that the model could predict these outbreaks, although the outbreaks report a small number of cases in many countries or states with heterogeneous characteristics.

**R:** Actually, our modelling framework is quite flexible and may be parameterized to reproduce different types of healthcare settings or wards (e.g. hemodialysis units), and epidemiological conditions. In this sense, it could be applied to assess the infectious risk and predict outbreaks in a wide array of situations, which is what we meant in this section of the discussion. The corresponding sentence has now been clarified as follows: “**Due to its flexibility, our model could simulate bloodborne pathogen transmission within a wide array of settings and thus** help predict such outbreaks, track past contamination events and assess the effectiveness of intervention measures.” (L577-578)

- I recommend reading literature on reprocessing and the use of single-use devices. https://www.who.int/publications/i/item/9789241549851, https://doi.org/10.1097/XEB.0000000000000146

**R:** We thank the reviewer for these references. They were added to the discussion section.

- I find your article very valuable and innovative. However, it is difficult to separate in practice the transmission of HCV, HBV, HIV through reprocessed medical devices from other bacterial and fungal hospital pathogens. Given that the reprocessing and reuse of devices is a frequent and heterogeneous practice in low-income countries, the model needs to be adjusted to include the specific risk for each device. I understand that the data from another publication by the same author has been used. However the categories of procedures are quite different

**R:** We thank the reviewer for his positive assessment of our article, and agree that our model could indeed also be used to study the transmission of bacteria and fungal pathogens, provided the parameters are adjusted accordingly.

Here we specifically take into account viral infection dynamics by using the duration of the eclipse phase for HCV and HBV, and using associated risks of transmission through medical procedures for these specific pathogens. We use estimated risks coming from a previously published risk-assessment paper which itself was based on a meta-analysis of hepatitis virus infection associated with different invasive procedures. For clarification, we have now also added this specific reference to the methods section. The procedures listed in the Ain Shams data were linked to the procedures listed in the meta-analysis, based on expert opinions, as explained in Table S2.

- The model has found that the greatest attribution of transmission is due to the endotracheal tube, however, I find this result without biological plausibility. Although I have searched, I have not found a single reported case of any of the blood pathogens that have been attributed to using a reused endotracheal tube. However, they are identified as real risks in the guidelines. Although reprocessing and sterilizing endotracheal tubes may be common in low-resource settings, it is unlikely to go unreported.

**R:** We thank the reviewer for this very useful comment which led us to reexamine our assumptions as follows.

Endotracheal intubation was performed in 70% of surgeries in our database. They were incorrectly systematically assigned to the “surgery” procedure even though “endotracheal intubation” was also recorded as a specific procedure. In our initial simulations, this led to endotracheal tubes being used twice in operations requiring intubation and once in operations not requiring intubation. In this revised version of our work, this error has been corrected, leading to a decrease in the average number of endotracheal tubes used per year from 57,357 to 23,831, much closer to the annual available quantity of 22,500 in the high-resource Ain Shams hospital. As a consequence, although reuse of this type of device still occurred in our simulations, this reduced the predicted annual cumulative incidence to an average of 1/100,000 in the high-resource scenario (from 4/100,000 originally) and 60/100,000 in the low-resource scenario (from 100/100,000). In addition, the most at-risk medical device in the low-resource scenario (Figure 3) has changed to intravenous cannula. However, endotracheal tubes remained the most at-risk device in the high-resource scenario, and the ranking of the most at-risk wards did not change in both scenarios. Our results regarding the impact of testing interventions also remained similar.

In addition, based on expert opinion, we considered the risk of bloodborne pathogen transmission to be similar for endoscopy and endotracheal intubation. As mentioned above, in our meta-analysis we found a significant association between HCV positivity and endoscopy, especially in countries with HCV prevalence (> 5%). This may have led us to further overestimate the risk associated with endotracheal tubes, although we do not have data to better estimate the per-procedure risk associated with intubation. This is now mentioned in the limitations section of the Discussion:

“On a related note, we assumed a similar risk of bloodborne pathogen transmission for endotracheal intubation and endoscopy, possibly leading us to overestimate the risk associated with endotracheal tubes.” (L525-528)

**Reviewer 2**

Henriot et al have developed an agent-based model of the transmission of blood-borne pathogens in hospital wards. This model includes a detailed treatment of device use, contamination, and disinfection, and movement between hospital wards based on data collected from a hospital in Egypt. The authors then use this model to estimate incidence in different settings, and to evaluate the effect of screening interventions, finding that incidence could potentially be quite high in low-resource settings but that entry- and ward-level screening could lead a large reduction in incidence.

- The strengths of this paper include the clear writing, visually appealing and easily understood figures, and the novel approach to an under researched area. The model itself is carefully designed and is explained well in the manuscript. Although it is quite complex, the complexity is justified and its parameters are largely well-informed by the data. There are no major weaknesses, but some minor weaknesses / issues include the lack of availability of the code and the lack of validation of model results with real data. I also think that the intuition behind the results could be explained a little more carefully for readers not familiar with this area (e.g. why the model predicts that some procedures / tools carry higher risk, and whether this is unexpected or not).

**R:** We thank the reviewer for this very positive and encouraging comment. We agree that there is a lack of validation of the model and that the code needs to be made available. Although we can validate the demographic dynamics part of the model using our dataset, we cannot compare the estimated cases with real epidemiological data, as in the IMMHOTHep study, we did not have information on patients' HCV positivity at discharge. Nonetheless, we have added 2 figures in the supplementary material comparing the distribution of the number of procedures provided by the model with the real distribution of the number of procedures given by our data, as well as a figure showing the distribution of patient lengths of stay in both cases (Fig S9 and S10). In addition, the C++ and R codes for the model and analyses have been made available on a GitHub repository.

We have also added a paragraph to the discussion to further comment on our findings regarding varying risks across procedures, devices and wards, illustrating how they may be explained. Notably, following a comment by Reviewer 1, we have now revised our assessment of endotracheal tubes, for which the initially estimated risk was overestimated in our analysis.

“On one hand, the incidence of infection observed in each ward depends on several factors, such as the prevalence on admission to these wards, the number of devices available (which determines how these devices are reused), the average number of procedures undergone by patients, and the specific risk of transmission during each of these procedures. In our case, it is not entirely surprising to find that the emergency ICU is one of the wards with the highest risk, as this risk seems to be driven by the high number of procedures performed on patients in this ward (around 33 procedures per patient, making it the ward with the highest number of procedures performed on patients). In addition, the risk of infection in the OR ward appears to be the result of the variety of procedures carried out there, for which there is often a lack of medical supplies.

On the other hand, the ranking of the most at-risk medical devices is strongly driven by the quantity of available equipment and their frequency of use within the hospital. Finding IV cannulas and lancets to be the most at-risk devices in the low-resource setting scenario is not very surprising as they are used in 19 and 23 different wards, and they are lacking in 68% and 40% of these wards, respectively. In addition, the fact that we found some devices to be more at-risk for transmission of HCV or HBV in the lower-resource setting scenario is due to the overall lower number of devices available in this scenario. Some devices ended up requiring reprocessing and reuse in the lower-resource setting scenario, but not in the higher-resource setting scenario.” (L499-517)

**Introduction**

• L53: please could you remove the sentence beginning “To our knowledge”, here and in other places, as it is impossible to verify these claims and they are unnecessary anyway.

**R:** Thank you, we have removed this mention across the article.

**Methods**

• In table 1B, please state the units of the eclipse phase duration

**R:** We have added these units as requested.

• L129: is there any chance that the alpha_ip will ever sum to greater than 1? e,g, if patients undergo multiple procedures

**R:** We thank the reviewer for this remark. Here, the sum could not be greater than 1 as patients cannot undergo multiple procedures at the same time in the model. We have added a sentence to clarify this: “Note that each row of this matrix sums to 1 and that a patient can only undergo one procedure at each time step.” (L153-155)

• L186: I’m not sure I understand this equation.
◦ Does it make sense to sum the probabilities of devices in the summation? Isn’t there a risk that P_{S→E} is greater than 1, e.g. if lots of contaminated devices are present in the ward, the probability of infection given contamination is high, or lots of devices are required in the procedure?
◦ Why isn’t the expression something like:
1 – product(1 – r_p * P(X^k))? I appreciate that this expression will be similar to yours for small probabilities, but that the probabilities are small is not clear to the reader at this point. So it might be worth clarifying in the text that the expression is an approximation and is appropriate because these probabilities are small. Unless I am misunderstanding something.

**R:** We thank the reviewer for this comment. Indeed, this probability could theoretically be greater than 1, although in practice this would never be achieved as it would require the use of a very large number of contaminated devices. We do however agree that the equation proposed by the reviewer is more correct mathematically, as notably it does bound the maximum probability to 1. We have changed this initial expression by the one proposed above. This change did not modify our results.

• I think the authors should endeavour to make the code available on GitHub, with synthetic data as the raw data cannot be shared.

**R:** We fully agree and have made our code available on GitHub, as mentioned above.

**Results**

• What is the intuition / reasoning behind which wards and which procedures caused the highest numbers of infections (Fig 2)?

**R:** The estimated number of cases per type of device depends on the risk of transmission during a procedure using a particular device (which was previously estimated), the frequency of use of that device, and the availability of that device, the latter two conditioning the reuse frequency.

This is now explained in a new paragraph in the Discussion, which provides the reader some insight into our main findings in terms of most at-risk wards and devices, as explained above.

• Why does the distribution of which tools have the biggest impact on incidence change between resource settings (Fig 3)?

**R:** We thank the reviewer for this question. The fact that we find some devices to be more at-risk for transmission of HCV or HBV in the lower-resource setting scenario is due to the overall lower number of devices available in this scenario. Some devices ended up requiring reprocessing and reuse in the lower-resource setting scenario, but not in the higher-resource setting scenario, while the individual risk of infection due to exposure to a contaminated device did not change, leading to a difference in device-associated risk distribution between the two scenarios. This is now explained in the new discussion section.

• I know it’s only two data points, but it seems perhaps misleading that for some simulations in high-resource settings, the intervention increases incidence, which seems implausible for this type of intervention. I presume that it’s because of differing stochastic events between paired simulations or something like that, but it makes me wonder if the results make the effect of the intervention appear more uncertain that they should. I wonder if there was a better way to pair simulations (e.g. using random seed, or ranking them by incidence), or perhaps to take an average across a (greater) number of simulations before comparing? Just some thoughts.

**R:** We thank the reviewer for this helpful suggestion. The simulations were rerun by adding the seed correspondence between the different scenarios. The updated figure does not feature increased incidences in the high-resource scenario anymore:

• Are there any data available on hospital incidence of HCV or HBV with which to (qualitatively) validate your results? It’s hard to know (for me at least) if what we are seeing in Figure 1 is plausible or not.

**R:** We thank again the reviewer for this comment. In our case, we would have needed HCV RNA screening at discharge to allow validation of our predictions, but to our knowledge this type of data is not available in the literature. However, as mentioned in our discussion, our estimates lie with the same order of magnitude than HCV infection incidence estimated in the Egyptian community.

**Discussion**

• L520: While I am sure this model could be applied, I’m not sure it is easy given the data requirements and complexity of the model – perhaps just remove the word ‘easily’

**R:** We agree and have removed the word “easily”.

• L521: Could you report computation times somewhere in the manuscript? (Apologies if I missed it)

**R:** We now report computation times at the end of the Methods section: “All simulations were distributed across 18 CPU threads (running at 4.8Ghz with 64Gb of RAM) using the R packages ‘parallel’ (14) and ‘doParallel’ (15). The average computation time for 100 simulations of a scenario was 25 minutes.” (L370-373)

• L536: I would reword this sentence – making WHO elimination targets more easily achieved is not a prevention measure, and anyway it’s not clear that this type of model could help achieving elimination targets without establishing the overall importance of hospital transmission compared to community transmission.

**R:** We agree with the reviewer that achieving WHO elimination targets is not a prevention measure, and that it is hard to actually predict to what extent controlling transmission within hospitals would help achieve these targets. We have rephrased this sentence in a more prudent manner: “It could help implement more efficient prevention measures in hospitals and, in turn, could contribute to achieving WHO HCV elimination targets, especially in Egypt where community transmission has been largely controlled.” (L577-579)
