# Supplementary material for: An agent-based model to simulate the transmission dynamics of bloodborne pathogens within hospitals
Source: PLoS Comput Biol. 2025 Feb 24;21(2):e1012850. doi: 10.1371/journal.pcbi.1012850 (PMC11882061; doi:10.1371/journal.pcbi.1012850)
Supplement: Attachment — Submitted filename: Responses_to_reviewers_Plos_CB_auresp_2.docx [file pcbi.1012850.s027.docx]

We thank the reviewer for his comment on the risk of transmission associated with endotracheal intubation. Our work’s main focus is to present an innovative modelling framework which may be parameterized to reproduce a wide array of epidemiological situations with varying risk levels. In this regard, reviewer 1’s remark prompted us to perform an additional sensitivity analysis to assess the effect of different levels of risk associated with endotracheal intubation on the outputs of our model. These new findings were presented in the Discussion section, given the lack of data in the literature on this specific risk, and additional figures were added as supplementary information. Our response to the reviewer’s remark is detailed below.

**Reviewer’s comment**

Just a last observation: you argue based on expert opinion that the risk of bloodborne pathogen transmission is similar for endoscopy and endotracheal intubation, but I find it weak:

Endoscopy procedure was identified as a low risk of HCV transmission 1.48(0.95−2.3) in previous studies. Endoscopy is an invasive procedure that uses a rigid or flexible probe and these procedures frequently are related with gastric erosions, ulcers, and/or bleeding. These probes are have holes and channels and since always are reused needs standardized protocols for cleaning, disinfection and chemical sterilization. Several reports of device-associated infection due to endoscopes are described in the literature.

In contrast, endotracheal intubation is an invasive procedure that uses a laryngoscope, and insertion of cuffed or non-cuffed endotracheal tubes. These devices has two or three holes and the contact with blood is minimal and can be related to oral trauma, mucosal tracheal erosions or infrequently by pulmonary bleeding. Again, no device-associated infections due to reprocessed endotracheal tubes are described or reported.

Please justify your expert opinion based on the available evidence and rationale.

Otherwise I suggest to adjust the model, e.g. adding the probability of bleeding while endotracheal tube is in place, time of exposure (in days where greater risk comes with more exposure).

**R :** We agree that the specific per-procedure risk associated with endotracheal intubation may have been overestimated in our main analysis, as already mentioned in the Discussion section. Indeed, there does not seem to be any studies in the literature reporting infection with blood-borne viruses after this procedure. Nevertheless, as you mentioned, the risk may not be zero, as blood contact is still possible during intubation. In order to assess the impact of the value of this specific risk on our conclusions, we performed additional simulations considering 2 scenarios of risk reduction compared to our baseline assumption of a risk similar to that of endoscopy: **i.** reducing the risk by 50% and **ii.** reducing it by 90%. Overall, these changes resulted in noticeable but small changes in the outputs of our model, as now mentioned in the Discussion section (L513-527):

“In fact, although blood contact may occur during this procedure (e.g. caused by oral or tracheal trauma), it could be minimal and we found no evidence in the literature of bloodborne infections following endotracheal intubation. To assess the effect of a lower assumed risk associated with this procedure on the results of our model, we examined reductions of 50% and 90% of the baseline risk. Overall, risk reductions led to slightly lower yearly cumulative incidences in both high- and low-resource settings (S11 Fig). In both reduction scenarios, in the high-resource setting, this had no effect on the ranking of the most at-risk devices, as the available quantity of endotracheal tubes was still largely insufficient compared to other devices (S12 Fig); the ward ranking also remained unchanged (S13 Fig). In the low-resource setting, endotracheal tubes became ranked as the 6th or 9th most at risk devices (compared to 4th in the baseline scenario) when the assumed per-procedure risk decreased by 50 or 90% (S12 Fig). Finally, the OR ward (in which most endotracheal intubations are performed) was estimated to be the third most at-risk ward rather than the second one in low-resource settings, with average reductions in yearly cumulative incidence of 31.9% and 59.4%, respectively (S13 Fig).”

We also added 3 supplementary figures describing the daily incidence rates (S11 Fig), yearly attributable portion to new cases for each device (S12 Fig), and ward-associated yearly cumulative incidence (S13 Fig), for each reduction scenarios for both high- and low-resource settings.
